# NLRP3 selectively drives IL-1β secretion by *Pseudomonas aeruginosa* infected neutrophils and regulates corneal disease severity

**DOI:** 10.1038/s41467-023-41391-7

**Published:** 2023-09-20

**Authors:** Martin S. Minns, Karl Liboro, Tatiane S. Lima, Serena Abbondante, Brandon A. Miller, Michaela E. Marshall, Jolynn Tran Chau, Alicia Roistacher, Arne Rietsch, George R. Dubyak, Eric Pearlman

**Affiliations:** 1 Departments of Ophthalmology and Physiology & Biophysics, University of California, Irvine, CA USA; 2 Department of Physiology & Biophysics, Case Western Reserve University, Cleveland, OH USA; 3 Department of Molecular Biology and Microbiology, Case Western Reserve University, Cleveland, OH USA; 4 Present Address: Odyssey Therapeutics, Boston, MA USA; 5 Present Address: Department of Biological Sciences, California State Polytechnic University, Pomona, CA USA

**Keywords:** Inflammasome, Bacterial infection

## Abstract

Macrophages infected with Gram-negative bacteria expressing Type III secretion system (T3SS) activate the NLRC4 inflammasome, resulting in Gasdermin D (GSDMD)-dependent, but GSDME independent IL-1β secretion and pyroptosis. Here we examine inflammasome signaling in neutrophils infected with *Pseudomonas aeruginosa* strain PAO1 that expresses the T3SS effectors ExoS and ExoT. IL-1β secretion by neutrophils requires the T3SS needle and translocon proteins and GSDMD. In macrophages, PAO1 and mutants lacking ExoS and ExoT (*ΔexoST*) require NLRC4 for IL-1β secretion. While IL-1β release from *ΔexoST* infected neutrophils is also NLRC4-dependent, infection with PAO1 is instead NLRP3-dependent and driven by the ADP ribosyl transferase activity of ExoS. Genetic and pharmacologic approaches using MCC950 reveal that NLRP3 is also essential for bacterial killing and disease severity in a murine model of *P. aeruginosa* corneal infection (keratitis). Overall, these findings reveal a function for ExoS ADPRT in regulating inflammasome subtype usage in neutrophils versus macrophages and an unexpected role for NLRP3 in *P. aeruginosa* keratitis.

## Abstract (web summary)

Bacterial infection of immune cells can result in engagement of different immunological pathways. Here the authors show that a *Pseudomonas aeruginosa* type three secretion system exoenzyme is linked to the differential selection of inflammasome usage between macrophages and neutrophils.

## Introduction

The discovery of the Gasdermin (GSDM) family of pore forming proteins has greatly increased our understanding of the mechanism of cell death in inflammation, cancer, and autoimmunity in addition to host defense, and has identified potential new targets for pharmacological intervention^1–3^. Specifically, Gasdermin D (GSDMD) mediates inflammasome-induced release of bioactive IL-1β and pyroptosis in myeloid leukocytes. Compared with macrophages, there are relatively few studies examining the role of GSDMD or other Gasdermin family members in neutrophils, even though neutrophils vastly outnumber other cells recruited to sites of inflammation and infection, especially at early time points. We and others reported that neutrophils are a major source of IL-1β in response to canonical NLRP3 activators such as ATP, to *Streptococcus pneumoniae* activation of NLRP3, or to *Salmonella* activation of NLRC4; further, in contrast to macrophages IL-1β secretion by neutrophils occurs in the absence of pyroptotic cell death^4–6^. We recently demonstrated that the underlying reason for the absence of pyroptosis in NLRP3 activated neutrophils is that N-GSDMD localizes to granules and autophagosomes rather than the plasma membrane, and that although IL-1β secretion is GSDMD dependent, there is an additional requirement for ATG7 in neutrophils that supports a role for autophagy associated secretion^7^. We also showed that N-GSDMD co-localizes with primary granules that leads to release of neutrophil elastase into the cytosol, which also cleaves pro-GSDMD^7^.

**Fig. 1:**
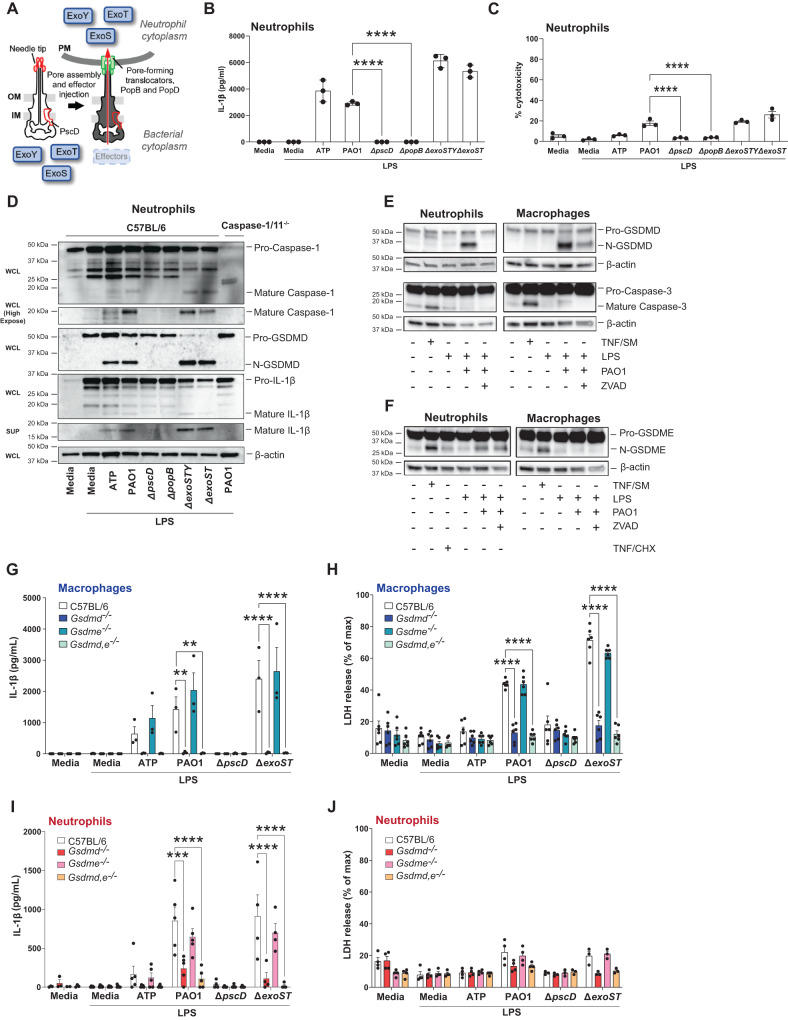
The role of T3SS, GSDMD and GSDME in IL-1β secretion by *P. aeruginosa* infected neutrophils. **A** *P. aeruginosa* T3SS is preassembled and spans the bacterial cell envelope (IM, inner membrane, OM, outer membrane and PG, peptidoglycan layer). Following cell contact, the pore-forming translocator proteins, PopB and PopD, assemble into a pore, which docks to the needle tip (PcrV, red). Effector secretion is triggered, resulting in export of effector proteins across the bacterial cell envelope and host cell plasma membrane (PM) into the cytosol of the targeted cell. *P. aeruginosa* strain PAO1 used in the current study expresses ExoS, ExoT, and ExoY, but not ExoU. **B** Quantification of IL-1β by ELISA, and **C** Cell death quantified by lactose dehydrogenase (LDH) activity in the supernatant of infected cells. **D** Caspase-1, GSDMD and IL-1β cleavage in whole cell lysates (WCL) and supernatants (SUP) from C57BL/6 and caspase-1/11^-/-^ bone marrow neutrophils primed 3 h with LPS and incubated 1 h with ATP or 1 h with *P. aeruginosa* strain PAO1 or PAO1 mutants ∆*pscD* (no needle), ∆*popB* (no translocon), ∆*exoSTY* or ∆*exoST*. **E**, **F** Caspase-3, GSDMD and GSDME cleavage in lysates with supernatants from C57BL/6 bone marrow neutrophils and bone marrow derived macrophages primed with LPS and infected with PAO1 in the presence of ZVAD. TNF-α and SMAC mimetic (TNF/SM) induced caspase-3 / GSDME cleavage after 4 h (no LPS priming). **G**–**J** IL-1β secretion and LDH release from infected bone marrow-derived macrophages and bone marrow neutrophils from C57BL/6, *Gsdmd*^-/-^ *Gsdme* ^-/-^ and *Gsdmd*/e^-/-^ mice primed with LPS and incubated with PAO1 or indicated mutants. Western blots are representative of 3 repeat experiments. Each data point represents one independent experiment (3-4 biological replicates); error bars are mean + /- standard deviation (SD). Statistical significance was assessed by 1-way ANOVA followed by a Brown-Forsythe post-test (B, C), or using 2-way ANOVA followed by Tukey’s post-test (G-J). For all figures, ****p < 0.0001, ***p < 0.001; **<0.01.

To determine if GSDMD is also required for IL-1β secretion by neutrophils infected with pathogenic bacteria, we examined the ubiquitous environmental Gram-negative bacterium *P. aeruginosa*, which is an important cause of dermal, ocular, and pulmonary disease, including cystic fibrosis. The Type III secretion system (T3SS) is a major virulence factor of *P. aeruginosa* that includes a complex needle structure that penetrates the plasma membrane of the target mammalian cell and injects effector proteins directly into the cytosol (Fig. 1A). Up to four effectors (exoenzymes) with distinct functions are injected via the T3SS: the phospholipase ExoU which induces cell lysis, and ExoS and ExoT which modulate protein function through their ADP ribosyl transferase (ADPRT) or GTPase activating protein (GAP) activities^8,9^. Most clinical isolates express ExoT, but for reasons not well understood, clinical isolates express either ExoS or ExoU, but rarely both exoenzymes^8,10,11^. ExoY is produced by most T3SS expressing strains and has adenylyl cyclase activity, but its role in infection remains uncertain^12–14^.

Our studies have focused on blinding *P. aeruginosa* infections of the cornea (keratitis), which are a major cause of corneal ulcers in the USA and worldwide, including a very recent outbreak of multi-drug resistant bacteria from commercial saline eyedrops^15–17^.Quantitative PCR analysis of patients with corneal ulcers caused by *P. aeruginosa* or *S. pneumoniae* revealed elevated gene expression of IL-1β, NLRC4, NLRP3 and ASC compared with uninfected human corneas associated with the infiltrate comprising >90% neutrophils^11^. We also used a murine model of *P. aeruginosa* keratitis and reported that an intact T3SS and the ADPRT activities of ExoS and ExoT are required for virulence in infected corneas^18^. More recently, we showed that ExoS ADP-ribosylates the RAS small GTPase in neutrophils and inhibits assembly of the NADPH oxidase complex, resulting in lower ROS production and impaired bacterial killing^19^.

Distinct roles for Gasdermin family proteins in neutrophils versus macrophages also applies to GSDME, which was first reported as a mutated gene (DFNA5) associated with hearing loss, but also forms plasma membrane pores following cleavage by caspase-3^1^. The *Yersinia* T3SS acetyltransferase *YopJ* activates GSDME rather than GSDMD via a RIPK1/caspase-3 pathway to induce IL-1β secretion and cell death in neutrophils^20^.

In the current study, we characterized the relative contributions of T3SS, the NLRP3 versus the NLRC4 inflammasomes, and GSDMD and GSDME to IL-1β release and pyroptosis in neutrophils versus macrophages following infection with *P. aeruginosa*. Surprisingly, we found that the ExoS ADP ribosyltransferase activity led to IL-1β secretion through the NLRP3 rather than the NLRC4 inflammasome in neutrophils, but not macrophages. Consistent with these in vitro findings, we identified a selective role for NLRP3 in regulating *P. aeruginosa* growth and disease severity in a murine model of blinding corneal infection.

Finally, although GSDMD is reported to mediate formation of neutrophil extracellular traps (NETosis) in response to PMA or intracellular LPS^21,22^, we found that GSDMD was not required for either reactive oxygen species (ROS) dependent phorbol myristate acetate (PMA) induced NETosis or ROS-independent NETosis induced by *P. aeruginosa*. Overall, these observations underscore how inflammasome signaling networks are differentially engaged by the *P. aeruginosa* T3SS in neutrophils versus macrophages and illustrate their importance in disease pathogenesis.

## Results

### P. aeruginosa induced IL-1β release by neutrophils is dependent on the T3SS needle and translocon and is mediated by GSDMD

Figure 1 A depicts the major proteins that comprise the *P. aeruginosa* T3SS, including the injectosome (the needle structure) and the translocon pore that penetrates the host cell membrane. These transport the effector exoenzymes from the bacterial cytosol through the inner and outer membranes and into the target eukaryotic cell. To examine the role of T3SS in IL-1β secretion, bone marrow neutrophils from C57BL/6 mice were isolated by negative bead selection (>95% purity), incubated 3 h with LPS to induce pro- IL-1β, and infected with *P. aeruginosa* strain PAO1 that expresses exoenzymes ExoS, T and Y, with ∆*pscD* (apparatus mutant), ∆*popB* (translocon mutant), or with exoenzyme mutants ∆*exoSTY* and ∆*exoST* that express the needle structure, but not the exoenzymes.

We found that only strains expressing the intact needle structure (PAO1, ∆*exoSTY* and ∆*exoST*) induced IL-1β secretion (Fig. 1B). LDH release, although higher in PAO1 infected neutrophils was <30% of the lysis buffer control (Fig. 1C**)**. Flagellin also induces IL-1β secretion by macrophages^23–25^; however, there were no significant differences between PAO1 and a ∆*fliC* mutant in the activation of IL-1β secretion or LDH release by neutrophils (Fig. S1A, B**)**. Notably, casein-elicited peritoneal neutrophils that were infected for 1 h with PAO1 or *P. aeruginosa* mutant strains in the absence of LPS priming released IL-1β with similar strain-dependent differences as was observed in LPS-primed bone marrow neutrophils (Fig. S1C, D).

Consistent with these data, pro-caspase-1, pro-GSDMD and pro-IL-1β were cleaved to the bioactive forms in neutrophils infected with *P. aeruginosa* strains PAO1, ∆*exoSTY* and ∆*exoST*, but not in neutrophils infected with the ∆*pscD* apparatus mutant or the ∆*popB* translocon mutant; caspase-1 and IL-1β were also detected in cell supernatants following TCA precipitation (Fig. 1D).

Canonical ATP - induced activation of NLRP3 and IL-1β secretion in neutrophils is dependent on GSDMD, which in contrast to macrophages, does not lead to accumulation of plasma membrane N-GSDMD pores or to pyroptotic cell death^7^. To determine the role of GSDMD and GSDME in *P. aeruginosa* – induced IL-1β secretion, bone marrow neutrophils and bone marrow derived macrophages from C57BL/6, *Gsdmd*^*-/-*^*, Gsdme*^*-/-*^ and *Gsdmd*^*-/-*^*/Gsdme*^-/-^ mice were primed for 3 h with LPS and infected with PAO1 strain for 45 min prior to analysis of pro-GSDMD and pro-GSDME cleavage. As a positive control for caspase-3 and pro-GSDME cleavage, neutrophils and macrophages were stimulated with TNF-α plus a second mitochondria-derived activator of caspases (SMAC) mimetic (SM), which activate the ripoptosome and apoptotic caspases that mediate GSDME cleavage^26^, or with TNF-α + cycloheximide (CHX) as described^20^. Cells were also stimulated in the presence of the pan-caspase inhibitor ZVAD, and macrophages were incubated in the presence of glycine to inhibit cell lysis. GM-CSF was added to neutrophil cultures to prevent spontaneous caspase-3 mediated apoptosis.

GSDMD was cleaved in neutrophils and macrophages following infection with PAO1 (Fig. 1E). Caspase 3 and GSDME were also cleaved in response to TNF/SM in neutrophils and macrophages and there was no effect of TNF/CHX; however, PAO1 infection resulted in GSDME processing in neutrophils, but not in macrophages (Fig. 1E, F). Notably, the PAO1-induced accumulation of cleaved GSDME in neutrophils was not inhibited by ZVAD. Lien et al. and Poltorak et al. showed that caspase-8 mediates GSDMD processing in *Yersinia* infected macrophages^27^,^28^, and Vince et al.^29^ recently reported that caspase-8 is associated with GSDMD cleavage in macrophages from patients with mutations in the X-linked inhibitor of apoptosis protein (XIAP) and in murine macrophages incubated with IAP antagonists. However, although caspase-8 was cleaved in TNF/SM treated macrophages and neutrophils, caspase-8 was not cleaved following PAO1 infection (Fig. S1E**)**, indicating that there is no requirement for caspase-8 in GSDMD processing or IL-1β secretion induced by PAO1 infection of neutrophils.

Taken together with the absence of caspase-3 processing by PAO1, these findings indicate that PAO1 infected neutrophils cleave pro-GSDME by a caspase-independent mechanism. This mechanism likely involves serine proteases such as Granzyme B, which is also produced by neutrophils^26,30^.

To ascertain if GSDMD and GSDME are required for IL-1β secretion following infection with ExoS expressing *P. aeruginosa*, bone marrow neutrophils isolated from *Gsdmd*^*-/-*^*, Gsdme*^*-/-*^ and *Gsdmd*^*-/-*^*/Gsdme*^*-/-*^ mice were primed with LPS and incubated 1 h with ATP, PAO1, ∆*pscd* or ∆*exoST* mutant bacteria. We found that IL-1β secretion and LDH release by macrophages stimulated with ATP, PAO1 or ∆*exoST* mutants were significantly lower in *Gsdmd*^*-/-*^ and *Gsdmd*^*-/-*^*/Gsdme*^*-/-*^ macrophages compared with C57BL/6 macrophages; however, there was no significant difference in IL-1β secretion between macrophages from C57BL/6 and *Gsdme*^*-/-*^ mice (Fig. 1G, H). Similarly, IL-1β secretion and LDH release by neutrophils simulated with ATP or infected with PAO1 or ∆*exoST* was completely dependent on GSDMD with no obvious contribution from GSDME **(**Fig. 1I, J**)**.

Collectively, these findings demonstrate that *P. aeruginosa* induces IL-1β secretion and low levels of cell death in neutrophils, both of which are dependent on expression of the needle structure and the translocon. As Δ*exoST* mutants induced similar responses to wild type PAO1, we initially conclude that T3SS exotoxins are not required for IL-1β release. Further, although GSDMD and GSDME are processed in PAO1 infected neutrophils and macrophages, and GSDMD is required for IL-1β secretion and LDH release by both myeloid cell types, there is no apparent role for GSDME in IL-1β secretion induced by T3SS expressing *P. aeruginosa*.

#### IL-1β secretion by PAO1 infected neutrophils is not dependent on NLRC4

In macrophages, IL-1β secretion and pyroptosis induced by the needle structure and flagella of *P. aeruginosa* and other Gram-negative bacteria is mediated by NLRC4^24,31,32^. As the *P. aeruginosa* needle structure is required for GSDMD cleavage and IL-1β secretion by neutrophils, we next examined if this is dependent on NLRC4. Bone marrow neutrophils and bone marrow derived macrophages from C57BL/6 or *Nlrc4*^-/-^ mice were LPS primed and infected with PAO1, ∆*pscD* or ∆*exoST*, and IL-1β and LDH were quantified as before.

**Fig. 2:**
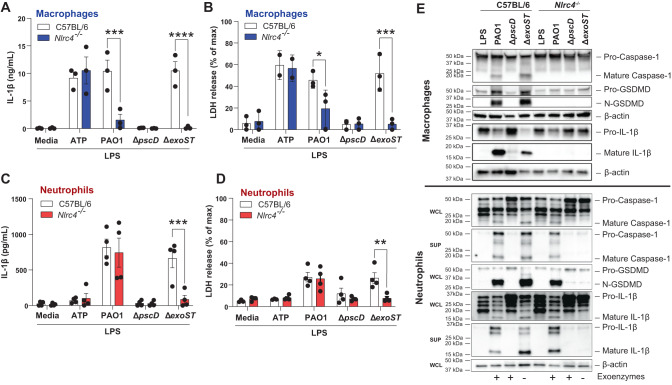
NLRC4 independent IL-1β secretion by PAO1 infected neutrophils. Bone marrow derived macrophages (**A**, **B**) and bone marrow neutrophils (**C**, **D**) from C57BL/6 and *Nlrc4*^-/-^ mice were primed 3 h with LPS and infected with PAO1, ∆*pscD* (no needle), ∆*popB* (no translocon), or ∆*exoST*. Quantification of IL-1β secretion by ELISA (**A**, **C**) and LDH release (**B**, **D**). **E** Caspase-1, GSDMD and IL-1β cleavage in infected macrophages and neutrophils from C57BL/6 and *Nlrc4*^-/-^ mice were examined by western blot. Macrophage westerns are combined whole cell lysate and TCA precipitated supernatants. Neutrophil whole cell lysates (WCL) and supernatants (SUP) were examined separately. Western blots are representative of 3 repeat experiments. Each data point in panels A-D represents one independent experiment (3-4 biological replicates). Statistical significance was assessed by 2-way ANOVA followed by Tukey’s post-test. Error bars are mean +/- SD.

IL-1β secretion and LDH release were elevated in C57BL/6 and *Nlrc4*^-/-^ macrophages stimulated with the canonical NLRP3 activator ATP; however, PAO1 and ∆exoST induced elevated IL-1β and LDH in C57BL/6, but not *Nlrc4*^-/-^ macrophages (Fig. 2A, B). Similarly, IL-1β secretion and LDH release by neutrophils infected with ∆*exoST* mutants was NLRC4 dependent; however, in marked contrast to macrophages, *Nlrc4*^*-/-*^ neutrophils infected with PAO1 secreted IL-1β and released LDH at levels not significantly different from C57BL/6 neutrophils (Fig. 2C, D).

Western blot analysis supported the ELISA findings. Caspase-1, GSDMD and IL-1β cleavage were visible in C57BL/6 macrophages infected with PAO1 or ∆*exoST* mutants but not in *Nlrc4*^*-/-*^ macrophages, indicating NLRC4 dependency (Fig. 2E, upper pane**l**). In contrast, cleaved caspase-1, GSDMD, and IL-1β were detected in both C57BL/6 and *Nlrc4*^*-/-*^ neutrophils infected with PAO1, indicating that this process is NLRC4-independent. However, cleaved caspase-1, GSDMD, and IL-1β were not detected in *Nlrc4*^*-/-*^ neutrophils infected with ∆exoST, indicating that this process is NLRC4 dependent. (Fig. 2E, lower panel).

Collectively, these findings are aligned with earlier reports that in macrophages, *P. aeruginosa* induced IL-1β secretion is dependent on NLRC4 inflammasome signaling^31,32^; however, we now show that in neutrophils infected with *P. aeruginosa* expressing ExoS and ExoT (PAO1), GSDMD cleavage and IL-1β secretion is NLRC4 independent.

#### NLRP3 dependent IL-1β secretion by *P. aeruginosa* infected neutrophils is regulated by ExoS ADPRT

As IL-1β is secreted by PAO1 infected neutrophils in the absence of NLRC4, we asked if ExoS and/or ExoT induce IL-1β secretion through the NLRP3 inflammasome. Bone marrow neutrophils and bone marrow derived macrophages from C57BL/6 and *Nlrp3*^-/-^ mice were infected with either PAO1 or the ∆*exoST* mutant strain, and IL-1β secretion, LDH release, and cleavage of pro- IL-1β and pro-GSDMD were assayed.

**Fig. 3:**
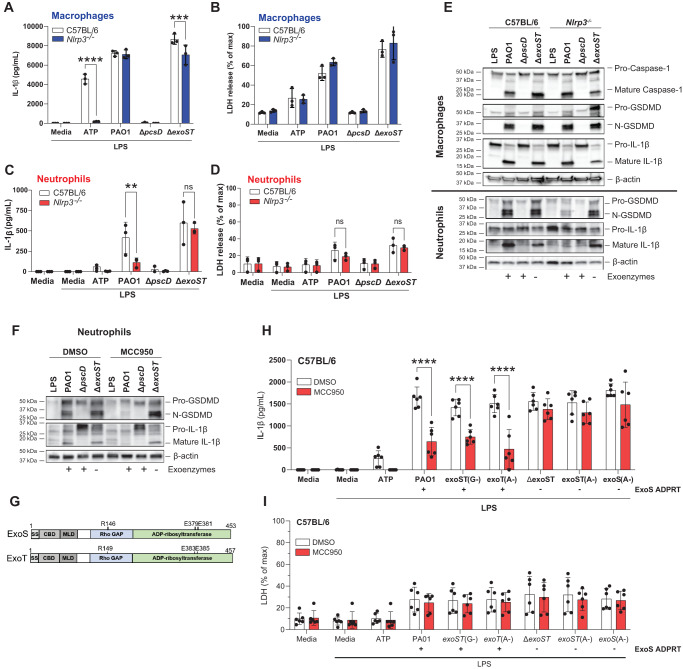
PAO1 mediated IL-1β secretion by neutrophils is NLRP3 dependent in the presence of ExoS ADPRT. Bone marrow derived macrophages (**A**, **B**) and bone marrow neutrophils (**C,** **D**) from C57BL/6 and *Nlrp3*^-/-^ mice were incubated with PAO1, ∆*pscD* or ∆*exoST*. IL-1β secretion and LDH release were quantified. **E** GSDMD and IL-1β cleavage was examined by western blot in macrophages and neutrophils from *Nlrp3*^-/-^ mice. **F** GSDMD and IL-1β cleavage in C57BL/6 neutrophils infected with PAO1 or mutants in the presence of the NLRP3 inhibitor MCC950. Protein cleavage was examined in lysates combined with TCA precipitated supernatants. **G** GTPase (GAP) and ADP ribosyltransferase (ADPRT) regions of ExoS and ExoT showing amino acids required for enzymatic function and which are also the sites of point mutations. **H**, **I** Neutrophils from C57BL/6 mice infected with PAO1, ∆*exoST* or mutants with indicated point mutations (ExoS, ExoT A-, G-) in the presence of MCC950. Strains expressing ExoS ADPRT are indicated. Western blots are representative of 3 repeat experiments. For panels **A**–**D**, H, I, each data point represents one independent experiments (3-6 biological repeats). Error bars are mean +/- SD. Statistical significance was assessed by 2-way ANOVA followed by Tukey’s post-test.

Consistent with the dominant role for NLRC4 in IL-1β secretion and LDH release by macrophages infected with PAO1 or the ∆*exoST* mutant, there was no difference in IL-1β secretion or LDH release between *Nlrp3*^-/-^ and C57BL/6 macrophages, although LPS/ATP induced IL-1β secretion was NLRP3 – dependent (Fig. 3A, B**)**.

In marked contrast to macrophages, IL-1β secretion by PAO1 infected *Nlrp3*^*-/-*^ neutrophils was significantly lower than in infected C57BL/6 neutrophils, whereas there was no difference between C57BL/6 and *Nlrp3*^*-/-*^ neutrophils infected with the ∆*exoST* mutant (Fig. 3C). As in prior experiments, LDH release from infected neutrophils was <30% of the detergent control (Fig. 3D). Consistent with the ELISA data, there was no difference in IL-1β, caspase-1 or GSDMD cleavage between C57BL/6 and *Nlrp3*^*-/-*^ macrophages infected with PAO1 or ∆*exoST* (Fig. 3E, upper panel), whereas IL-1β and GSDMD cleavage was lower in *Nlrp3*^-/-^ neutrophils infected with PAO1, but not the ∆exoST mutant (Fig. 3E, lower panel).

As a second approach to examine the role of NLRP3, neutrophils from C57BL/6 mice were incubated with the well-characterized, highly specific NLRP3 inhibitor MCC950^33^ prior to infection with *P. aeruginosa*. As with *Nlrp3*^-/-^ neutrophils, GSDMD and IL-1β cleavage was inhibited in MCC950 treated neutrophils following infection with PAO1, whereas there was no effect of MCC950 in neutrophils infected with the ∆*exoST* mutant (Fig. 3F). *Nlrc4*^*-/-*^ neutrophils incubated with MCC950 responded the same as C57BL/6 neutrophils, i.e., MCC950 inhibited IL-1β following infection with PAO1 or ExoS ADPRT expressing mutants (Fig. S2A, B). Collectively, these findings indicate that ExoS and/or ExoT either drive NLRP3 inflammasome usage or inhibit NLRC4 activity in neutrophils, but not in macrophages.

*P. aeruginosa* ExoS and ExoT are bifunctional exoenzymes as shown in Fig. 3G—a GTPase Activating Protein (GAP) domain that targets small GTPases (Rho, Rac, CDC42) and disrupts host cell cytoskeletal activities, and an ADP ribosyltransferase (ADPRT) that post-translationally modifies the activity of multiple proteins^8^. To identify which of these functions of ExoS or ExoT mediate NLRP3 dependent IL-1β secretion, bone marrow neutrophils from C57BL/6 mice were infected with PAO1 expressing point mutations in the enzymatic regions of either the GAP or ADPRT domains of ExoS or ExoT (indicated in the diagram) in the presence or absence of MCC950.

We found that point mutations in GAP domains of ExoS and ExoT (*exoST(G-)*) or in the ExoT ADPRT domain (*exoT(A-)*) phenocopied PAO1 in showing MCC950 inhibition (NLRP3 activity), whereas point mutations inactivating both ExoS and ExoT ADPRT domains (exoST(A-)), or only the ExoS ADPRT domain (exoS(A-)) phenocopied the ∆*exoST* mutant strain in showing no effect of MCC950 on readouts of inflammasome activity (Fig. 3H, I). Consistent with a role for ExoS rather than ExoT in licensing NLRP3 rather than NLRC4 usage, IL-1β secretion by neutrophils infected with ∆*exoT* mutants (ExoS expressing) was dependent on NLRP3, whereas IL-1β secretion following infection with ∆*exoS* mutants (ExoT expressing) was not inhibited by MCC950 (Fig. S2G, H).

Taken together, these findings identify a requirement for the ADP ribosyl transferase activity of ExoS to redirect inflammasome signaling to NLRP3-dependent rather than NLRC4-dependent pathways during *P. aeruginosa* infection of neutrophils.

In contrast to our findings, a recent study by Santoni et al. showed that neutrophil IL-1β secretion and LDH release following infection with PAO1 was completely dependent on NLRC4^34^. Using their protocol (3 h infection, no LPS priming), we also found that IL-1β secretion by PAO1 is NLRC4-dependent whereas our protocol (3 h priming, 1 h infection) is NLRC4 independent (Fig. S2C-F). We reported that NLRP3 is transcribed de novo in neutrophils in response to NFκB-activating mediators^4^; it is therefore likely that in the absence of priming by LPS, a 3 h PAO1 infection at a 10:1 multiplicity of infection does not induce sufficient NLRP3 expression in bone marrow neutrophils, leaving NLRC4 as the default inflammasome sensor responding to *P. aeruginosa*. The primary role for NLRP3 is supported by our findings that IL-1β secretion by peritoneal neutrophils in the absence of LPS priming is also NLRP3 dependent (Fig. S2E, F). The latter model can be considered more physiological because blood neutrophils recruited into the inflammatory peritoneal environment are already primed and are expressing NLRP3.

#### *P. aeruginosa* corneal infection (keratitis) is regulated by NLRP3 and Caspase-1

As noted earlier, *P. aeruginosa* is an important cause of painful corneal infections worldwide, resulting in visual impairment and blindness. To characterize the relative contribution of NLRP3 and NLRC4 in vivo, we used a well-established murine model of *P. aeruginosa* keratitis in which the corneal epithelium is abraded, and 5×10^4^ GFP expressing bacteria (GFP-PAO1) are added topically in 2 µl saline^35^. In this model of PAO1 infected C57BL/6 corneas, early central corneal opacification is associated with neutrophil infiltration resulting in control of bacterial growth; however, in the absence of IL-1β, bacteria replicate in the central cornea (at the site of infection), leading to perforation within 48-72 h post-infection^35^.

To determine the relative contribution of NLRP3 and NLRC4 inflammasomes in *P. aeruginosa* keratitis, corneas of *Nlrp3*^-/-^ and *Nlrc4*^-/-^ mice, and C57BL/6 mice given systemic MCC950 were infected with PAO1, and corneal opacification, GFP intensity and CFU were examined after 24 h.

**Fig. 4:**
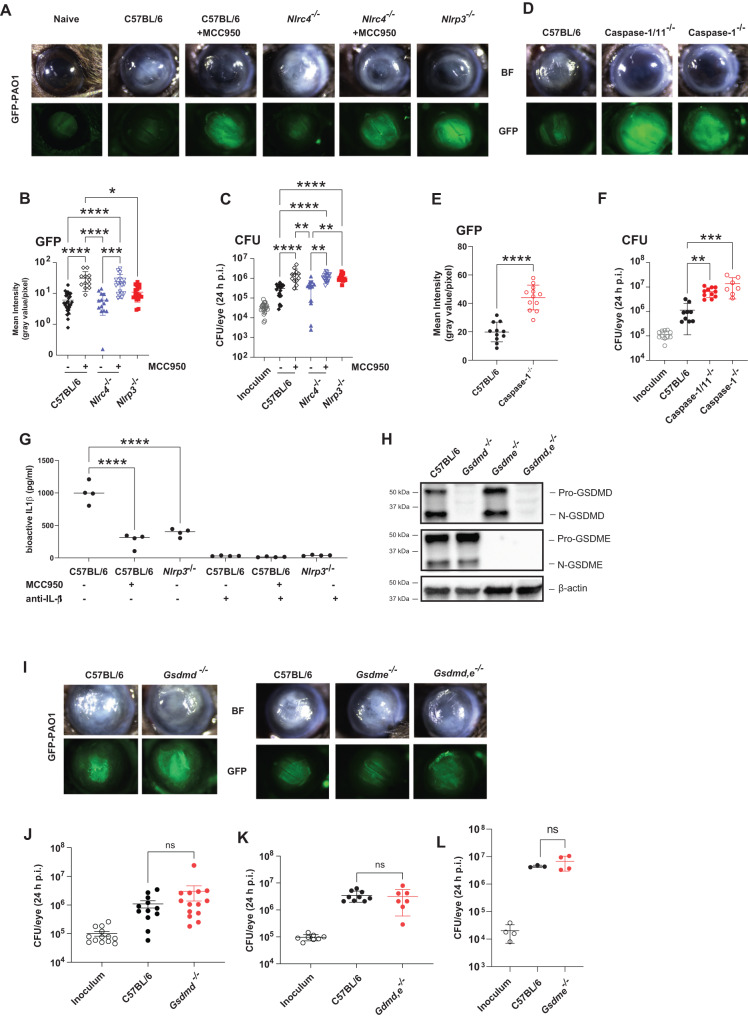
The role of inflammasomes, caspase-1, GSDMD and GSDME in *P. aeruginosa* corneal infections. **A-C** Corneas of MCC950 treated C57BL/6, *Nlrc4*^*-/-*^, and *Nlrp3*^*-/-*^ mice were infected with 5×10^4^ GFP expressing PAO1. After 24 h, corneal opacification and total GFP bacteria were quantified by image analysis, and viable bacteria were measured by CFU. **A** representative images of infected corneas and GFP-PAO1. **B** Quantification of GFP in infected corneas. **C** CFU in infected corneas. **D**–**F** Corneal opacification, GFP-PAO1 and CFU in C57BL/6, *caspase-1 and caspase-1/11*^-/-^ mice. **G** Bioactive IL-1β in infected corneas from C57BL/6, MCC950 treated mice, and *Nlrp3*^*-/-*^ mice using the IL-1R reporter cells in the presence of neutralizing anti-IL-1β **H**–**L** PAO1 infected C57BL/6, *gsdmd*^-/-^*, gsdme* ^-/-^ and *gsdmd* / *gsdme* ^-/-^ corneas. **H** GSDMD and GSDME cleavage in infected corneas. **I** Representative corneas, and CFU (**J–L**) GSDMD and GSDME cleavage products were examined by western blot. Western blots are representative of 3 repeat experiments. Each data point represents a single infected cornea from 3 independent experiments with 4 infected corneas. Statistical significance was assessed by 1-way ANOVA followed by Kruskal-Wallis post-test for in vivo analysis. **** represents *p* < 0.0001, *** is *p* < 0.001; ** is <0.01, * is <0.05.

Infected *Nlrc4*^-/-^ mice exhibited central corneal opacity and low levels of GFP, similar to infected C57BL/6 corneas; however, when these mice were given systemic MCC950, there was less corneal opacification and increased GFP-bacteria in the central cornea and significantly elevated CFU (Fig. 4A–C**)**. *Nlrp3*^-/-^ mice had the same phenotype as C57BL/6 and *Nlrc4*^-/-^ mice given MCC950, and there were no statistically significant differences in GFP or CFU between infected C57BL/6 and *Nlrc4*^-/-^ corneas. *Caspase-1/11*^*-/-*^ and *Caspase-1*^*-/-*^ mice had more severe corneal disease and significantly elevated GFP and CFU compared with C57BL/6 mice (Fig. 4D–F**)**. Infected corneas from MCC950 treated C57BL/6 mice and *Nlrp3*^-/-^ mice had significantly less bioactive IL-1β than untreated C57BL/6 corneas (Fig. 4G), although there was no difference in the number of neutrophils or monocytes in infected corneas of C57BL/6 compared with *Nlrp3-/-* mice (Fig. S3A–C).

Corneal disease and bacterial killing were also assessed in infected *Gsdmd*^-/-^*, Gsdme* ^-/-^ and *Gsdmd*^-/-^/*Gsdme* ^-/-^ mice. We found that GSDMD and GSDME were cleaved in infected corneas (Fig. 4H**)**; however, there were no significant differences in CFU or corneal disease between infected C57BL/6 and *Gsdmd*^-/-^*, Gsdme*^-/-^ or *Gsdmd*^-/-^/*Gsdme*^-/-^ mice (Fig. 4I–L**)**. There were also no differences in IL-1β secretion or the number of neutrophils and monocytes in infected corneas of C57BL/6 and *Gsdmd*^-/-^ mice (Fig. S3D–G). Finally, we found no role for neutrophil elastase in this process as infected *Elane*^-/-^ corneas showed no difference in corneal opacification, CFU or GFP compared with C57BL/6 mice (Fig. S3H–J**)**.

Overall, these findings support a role for NLRP3 and caspase-1 in regulating bacterial killing and corneal disease severity with no apparent role for NLRC4 and no requirement for GSDMD, GSDME, or neutrophil elastase.

#### Induction of neutrophil extracellular traps (NETs) by *P. aeruginosa* is not dependent on GSDMD or GSDME

As there was no role for GSDMD or GSDME in *P. aeruginosa* keratitis, and as these bacteria induce NETs in murine models of pulmonary and corneal infections^30,31^, we examined if this is related to NETosis induced by *P. aeruginosa*. NETosis is a coordinated mode of cell death in neutrophils that is associated with regulated release of nuclear DNA, histones and cytosolic proteins as neutrophil extracellular traps in response to phorbol myristate acetate (PMA) mediated protein kinase C (PKC) activation, or after incubation with bacteria or fungi^36,37^. NET proteins include histones, neutrophil proteases, and peptides that have antimicrobial and cytotoxic activities. GSDMD was reported to mediate NETosis induced by PMA^22^, or infection with intracellular *Salmonella, Citrobacter* or transfection with LPS that accumulates in the cytosol with consequent activation of non-canonical caspase-11 (caspases 4 and 5 in human cells)^21^.

**Fig. 5:**
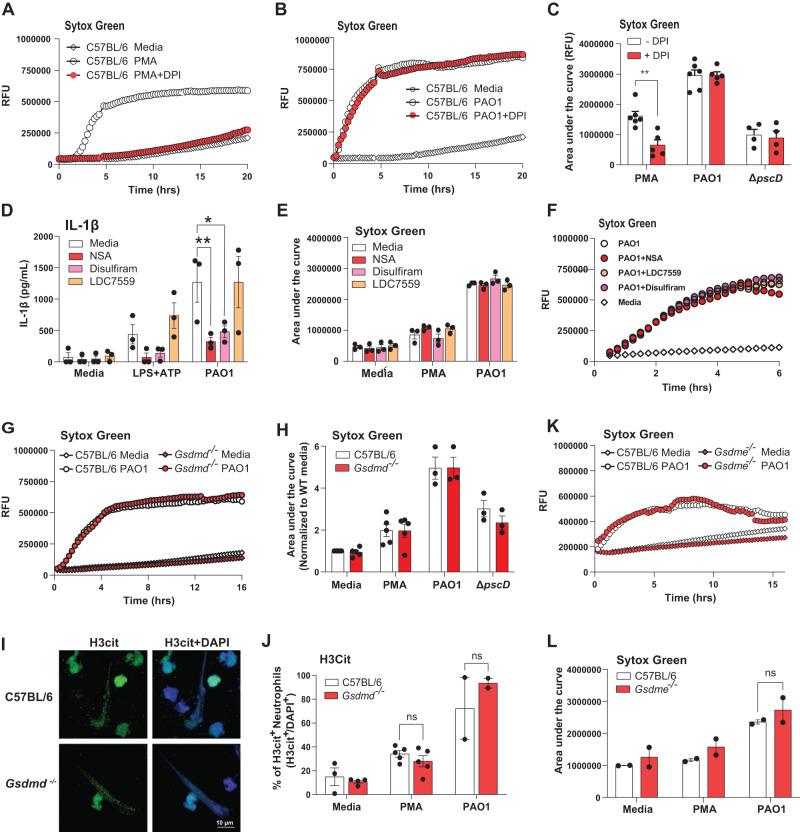
PAO1 induced NETosis is independent of ROS, GSDMD and GSDME. **A–C** Role of ROS in PMA stimulated, but not PAO1 infected peritoneal neutrophils. Representative time courses (**A, B**) and combined data from biological replicates (**C**). **D-F** IL-1β secretion (**D**) and NETosis (**E**) by peritoneal neutrophils incubated with reported GSDMD inhibitors necrosulfonamide (NSA), disulfiram, and LDC7559. NETosis was quantified by Sytox detection of extracellular DNA after 16 h (Area under the curve). Panel **F** is a representative time course. **G**–**J** Peritoneal neutrophils from C57BL/6 and *Gsdmd*^-/-^ mice incubated 16 h with PMA, PAO1 or ∆*pscD* and extracellular DNA measured by Sytox. **G** representative time course, and **H** quantification of the area under the curve. **I** Representative images of citrullinated histone 3 (H3Cit) as an indicator of NETosis in PAO1 infected neutrophils (original and other images are in Fig. S4F). **J** Quantification of H3Cit+ neutrophils per high power field. **K**, **L** Representative time course and quantification of NETosis in C57BL/6 and *Gsdme*^-/-^ mice. ** is <0.01, * is <0.05. Each data point represents one independent experiment (2-6 biological repeats). Error bars are mean +/- SEM. Statistical significance was assessed by 2-way ANOVA followed by Tukey’s post-test.

We found that NETosis induced by PMA and quantified by Sytox Green is dependent on production of reactive oxygen species (ROS) and can be inhibited with Diphenyleneiodonium chloride (DPI) as reported^38^; however, NETosis induced by PAO1 was not inhibited by DPI (Fig. 5A–C**)**, indicating that NETosis induced by PAO1 is ROS independent. This is consistent with our findings that ExoS ADPRT inhibits NADPH oxidase assembly and ROS production^19^.

To examine the role of GSDMD in NETosis induced by ExoS expressing *P. aeruginosa*, neutrophils isolated from the peritoneal cavity of C57BL/6 mice following sterile inflammation were incubated with PAO1 at a ratio of 30:1 in the presence of LDC7559, which was reported to inhibit GSDMD-dependent NETosis^22^, or with two other known GSDMD inhibitors, necrosulfonamide (NSA) and disulfiram^32,33^. We found a significant reduction in IL-1β secretion by neutrophils incubated with NSA or disulfiram, but not with LDC7559 (Fig. 5D**)**; however, there was no effect on NET formation (as quantified by accumulation of extracellular Sytox Green-DNA fluorescent complexes) by any of these compounds (Fig. 5E, F**)**.

As a second approach to examine the role of GSDMD in *P. aeruginosa* - mediated NETosis, peritoneal neutrophils isolated from C57BL/6 and *Gsdmd*^-/-^ mice were infected with PAO1, and NETosis induced by *P. aeruginosa* was quantified using either Sytox Green, or antibodies to citrullinated histone 3 (H3Cit). H3Cit is generated by protein arginine deiminase 4 (PAD4), which contributes to histone citrullination and chromatin decondensation during NETosis ^39,40^. We found no difference in Sytox Green signals from PMA stimulated or PAO1 infected neutrophils from C57BL/6 compared with *Gsdmd*^-/-^ mice, although there was less NETosis in neutrophils infected with the ∆*pscD* injectosome or the ∆*fliC* flagellin mutants (Fig. 5G, H**;** Fig. S4A, B**)**.

In agreement with the Sytox data, we found that up to 80% neutrophils were H3Cit+ following stimulation with PMA or infection with *P. aeruginosa* strain PAO1; however, there was no significant difference in H3Cit between neutrophils from C57BL/6 and *Gsdmd*^-/-^ mice (Fig. 5I, J, Fig. S4C**)**. There were also no differences in PAO1 induced NET formation by neutrophils from *Gsdme*^-/-^ and C57BL/6 mice (Fig. 5K, L**)**.

Overall, these observations clearly demonstrate using genetic and pharmacological approaches that neither GSDMD nor GSDME are required for NETosis induced by PMA or following infection with T3SS expressing *P. aeruginosa*. Rather than inhibit NETosis, we found that LDC7559 inhibited PMA - induced neutrophils ROS production (Fig. S4D, E**)**, which is consistent with its reported effect on NADPH oxidase^41^.

## Discussion

Bacterial T3SS effectors are efficiently injected into target eukaryotic cells where they can compromise the host immune response. For example, *Shigella flexneri* T3SS ubiquitin ligase IpaH7.8 selectively targets human GSDMB and GSDMD for proteasomal degradation, thereby inhibiting both GSDMB mediated bacterial killing and GSDMD mediated host cell lysis to protect the replicative environment of the bacteria^42,43^. *Pseudomonas, Salmonella*, and *Yersinia* T3SS and *Legionella* Type IV secretion activate IL-1β secretion and pyroptosis in murine macrophages primarily through recognition of needle structure proteins by NAIP1 and NAIP2 (humans have a single NAIP), for activation of the NLRC4 inflammasome^44^. Also, *Yersinia YopJ* acetyltransferase promotes macrophage pro-IL-1β and GSDMD cleavage and pyroptosis^27,28^.

In contrast to macrophages, there are relatively few reports on the role of neutrophils as a source of IL-1β during bacterial infection. In *Burkholderia* infected neutrophils, IL-1β secretion and pro-GSDMD cleavage by neutrophils requires caspase-1 or caspase-11, and selective deletion of caspase-11 in neutrophils results in impaired bacterial killing^45^. Further, survival of mice infected with *YopJ* expressing *Yersinia* is dependent on GSDME rather than GSDMD, and GSDME is selectively required for IL-1β secretion by neutrophils^20^. Consistent with that report, we found that *P. aeruginosa* induces pro-GSDME cleavage in neutrophils, though not in macrophages; however, IL-1β secretion was dependent on GSDMD and not GSDME. Further, while pro-GSDME is known to be cleaved by caspase-3^15^, we found that pro-caspase-3 was not cleaved in *P. aeruginosa* infected neutrophils. As we and others reported that serine proteases such as neutrophil elastase can cleave pro-GSDMD^7,22,46^, it is possible that serine proteases also cleave pro-GSDME in neutrophils. In support of this concept, Granzyme B released from natural killer or cytolytic T cells directly cleaves GSDME to drive lytic tumor cell death, and TLR4-activated neutrophils upregulate expression of the granzyme B serine protease^26,30^.

**Fig. 6:**
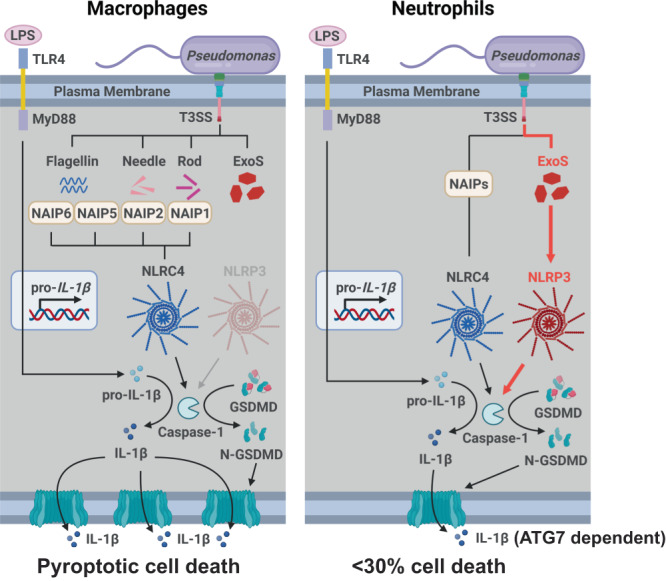
Predicted sequence of events in *P. aeruginosa* induced IL-1β secretion by macrophages and neutrophils. See text for description. Created with BioRender.com.

The differences in IL-1β secretion between macrophages and neutrophils infected with ExoS expressing *P. aeruginosa* are summarized in Fig. 6. Pre-incubation with LPS induces expression of NLRP3 and pro- IL-1β in macrophages and neutrophils; however, following macrophage infection with T3SS expressing *P. aeruginosa*, the needle and translocon structures selectively activate NLRC4 (through NAIP1 and NAIP2), resulting in caspase-1 mediated IL-1β and GSDMD cleavage, plasma membrane pore formation, pyroptosis and secretion of bioactive IL-1β, which is consistent with earlier reports on the role of NLRC4 in *P. aeruginosa* infected macrophages^31,32,47^. In contrast, we found that the needle structure can activate NLRC4 in neutrophils in the absence of exoenzymes (in ∆*exoST* mutants), leading to cleavage of GSDMD and IL-1β. However, in the presence of ExoS and ExoT, NLRP3 rather than NLRC4 was required for pro-GSDMD and IL-1β cleavage. We identified ExoS ADPRT as the mediator of selective NLRP3 activation. Using the NLRP3 inhibitor MCC950, *Nlrp3*^*-/-*^ mice, and *Nlrc4*^*-/-*^ mice, we also demonstrate that NLRP3 rather than NLRC4 is required for bacterial killing and preventing corneal perforation in *P. aeruginosa* keratitis.

Collectively, these findings demonstrate a physiological role for NLRP3 and caspase-1 in regulating infection in a clinically relevant murine model of blinding corneal disease. In contrast to these observations, Santoni et al. reported that IL -1β secretion and pyroptosis in PAO1 infected neutrophils was strictly dependent on NLRC4^34^. Although we were able to reproduce their observations using bone marrow neutrophils that were not primed with LPS prior to infection, we reported that NLRP3 expression in neutrophils needs to be induced by TLR priming^4^. In support of this, IL -1β secretion by casein - induced peritoneal neutrophils infected for 1 h with PAO1 was completely dependent on NLRP3 in the absence of LPS priming due to the in vivo priming that occurs as recruited neutrophils accumulate in the inflammatory milieu of the peritoneum. Also, in *P. aeruginosa* infection, NLRP3 and caspase-1, but not NLRC4 were required for IL -1β production, bacterial killing and corneal disease severity. While Santoni and colleagues showed a role for caspase-1 in a *P. aeruginosa* lung infection model, they did not examine if there is a role for NLRC4 in vivo^34^. We therefore conclude that selective usage of NLRP3 more closely resembles the activation state of neutrophils during infection and inflammation.

We also examined the role of GSDMD and GSDME during infection. Although GSDMD and GSDME were cleaved during corneal infection with PAO1, there was no effect of GSDMD and/or GSDME deficiency in on the progression of corneal disease or on bacterial killing. While we have yet to identify the underlying mechanisms, it is likely that it is due to GSDMD independent IL-1β secretion by neutrophils at later time points in vitro and in *Salmonella typhimurium* infected mice^48^.

The underlying mechanisms that regulate inflammasome dependence between neutrophils and macrophages have yet to be defined. While we have yet to identify the molecular target of ExoS ADPRT activity, we provide new data showing that macrophages make more NLRP3 on a per cell basis than neutrophils **(**Fig S1F**)**. This is consistent with reports by Schroder et al. showing more caspase-1 and ASC in macrophages compared with neutrophils on a per cell basis^49^. Aachoui et al. also showed higher production of NLRC4 in macrophages compared with neutrophils^50^. These findings support the concept that if NLRP3 or NLRC4 are substrates for ExoS ADPRT, the lower protein levels in neutrophils would result in a higher enzyme: substrate ratio that is consistent with ExoS having an observable effect in neutrophils compared with macrophages. Alternatively, as there is less NLRC4 in neutrophils than macrophages, ExoS ADP ribosylation leading to loss of function is more likely to be detected in neutrophils. Although these scenarios have yet to be tested, gain-of-function gene mutations in NLRP3 that cause cryopyrin associated period syndromes (CAPS) have been well described^51^. Further, neutrophils are the major source of IL-1β in CAPS patients, and gain-of-function mutations expressed only in neutrophils are sufficient to induce CAPS like symptoms in mice^52^.

NLRP3 activity is also regulated by post-translational modifications, including JNK phosphorylation of NLRP3, which potentiates oligomerization during LPS priming^53^. Enzymes that regulate ubiquitylation and deubiquitylation also regulate NRLP3 activity^54,55^. Additionally, the *Mycoplasma pneumoniae* community-acquired respiratory distress syndrome (CARDS) toxin ADP ribosylates NLRP3 to enhance inflammasome activity^56^. In support of this, macrophages incubated with recombinant CARDS toxin induced NLRP3-mediated IL-1β secretion via a mechanism requiring the ADPRT enzymatic activity of CARDS, and CARDS toxin interaction with NLRP3^57^, implying that ADP ribosylation resulted in increased NLRP3 activity. Similarly, Group A Streptococcus *SpyA* ribosyl transferase toxin induced NLRP3/caspase-1 dependent IL-1β secretion by macrophages, although these investigators did not examine inflammasome usage^58,59^. It is also plausible that effectors are translocated at different levels into macrophages and neutrophils. Rietsch and colleagues demonstrated hyper-translocation of ExoS into macrophages, which was associated with phagocytosis and loss of feedback inhibition^60^. However, given that we do not see an effect of NLRP3 in macrophages, we suspect that the differences are due to reduced substrate levels in neutrophils.

Finally, it will be important to consider how additional intracellular signals triggered by PAO1 infection of neutrophils might contribute to NLRP3 activation. Potential signals are increased K^+^ efflux via the translocon pores^61,62^ and accumulation of phosphatidylinositol-4-phosphate in the endosomal or dispersed trans-Golgi vesicles that recruit NLRP3 for subsequent trafficking to the NEK7-containing microtubule organizing centers where active NLRP3 inflammasomes are assembled^20,63,64^.

An additional mechanism for the PAO1 induced redirection from NLRC4 to NLRP3 inflammasome usage is that ExoS ADPRT inhibits NAIP or NLRC4, which remain inactive even in the absence of NLRP3. The possibility that ExoS ADPRT inhibits NLRC4 inflammasome signaling is supported by Santoni et al. who observed that IL-1β secretion was markedly increased in neutrophils infected with ExoS-deficient strains versus PAO1^34^. Although most reports cite modifications that enhance rather than inhibit NLRC4 activity^52,53^, ExoT was recently found to inhibit NLRC4 activation by blocking Crk2/PKCδ – mediated phosphorylation of NLRC4, which is required for activation^65^. Future studies will identify inflammasome proteins that are substrates for ExoS ADPRT. An important caveat in performing these experiments is that ExoS ADPRT is highly promiscuous compared with as it has multiple substrates compared with ExoT ADPRT^8^. In support of this concept, a recent report showed that expression of ExoS ADPRT correlated with decreased lytic cell death in corneal epithelial cells^66^, although the target proteins were not identified.

We reported that activation of the NLRP3 inflammasome in neutrophils by *Streptococcus pneumoniae* does not lead to pyroptotic cell death as quantified by LDH release^4,5^. Here we find that T3SS expressing *P. aeruginosa* induce low levels of LDH release (~30% of triton control) from LPS primed neutrophils compared with up to 80% macrophages under the same conditions, suggesting that this level of neutrophil pyroptosis may have only marginal physiological significance.

In addition to its role in mediating secretion of IL-1β and pyroptotic death, GSDMD has been linked to the release of neutrophil extracellular traps (NETs)^21,22^. However, more recent studies have questioned the requirement for GSDMD in ROS-dependent NET release and NETosis^34,67,68^. Although we found that in contrast to PMA, *P. aeruginosa* - induced NETosis was ROS independent, those reports are in agreement with results of the current study wherein GSDMD and GSDME had no apparent effect on NETosis induced by PMA or *P. aeruginosa* when NETs were quantified by Sytox staining of released DNA or by accumulation of H3Cit. There was also no difference in NETosis using *Gsdmd*^*-/-*^ or *Gsdme*^*-/-*^ neutrophils, or neutrophils treated with known GSDMD small molecule inhibitors. The absence of a role for GSDMD is consistent with our findings that N-GSDMD does not localize to the plasma membrane^7^, and may instead be mediating DNA and histone release from the nucleus into the cytoplasm as reported by Santoni et al.^34^. Overall, the underlying mechanisms of NETosis depend on the model of induction and the time course, and at present there is no settled consensus^39^.

In an earlier report, we showed that PAO1 infection of peritoneal neutrophils induced release of IL-1β that was attenuated (~50-60%) but not completely suppressed in neutrophils from *caspase-1*^-/-^ and *Nlrc4*^-/-^ mice^69^. In that study, production of mature IL-1β and bacterial killing in corneal infections were not inhibited in the absence of caspase-1 or NLRC4; rather, it was predominantly mediated by neutrophil elastase^69^. In the current study, we repeated the infection studies in neutrophil elastase (*Elane*)^-/-^ mice with PAO1 and found no differences in CFU or corneal disease severity. While this disparity is puzzling, we examined whether differences in the culture media, which has a profound effect on production of the T3SS^70^ (Fig. S4F), can explain this discrepancy but found no evidence to support this explanation. Santoni et al. also found no role for neutrophil serine proteases in IL-1β secretion^34^.

Overall, results from the current study reveal a previously unappreciated role for specific proteins of the bacterial T3SS in selectively directing activation of the NLRP3 versus NLRC4 inflammasomes in neutrophils but not macrophages. The findings also increase our understanding of how GSDMD versus GSDME cleavage is differentially utilized for IL-1β secretion and regulated cell death responses in neutrophils compared with macrophages, and in bacterial infections where neutrophils are the predominant cell type.

## Methods

### Ethics

All studies described have been approved by UC Irvine oversight committees, including the use of *Pseudomonas aeruginosa* by the UC Irvine Institutional Biosafety Committee, the use of mice as a source of neutrophils and macrophages and the murine model of corneal infection by the UC Irvine IACUC. The use of peripheral blood from healthy volunteers was approved by the UC Irvine Institutional Review Board.

### Reagents

All reagents and antibodies are listed in Supplementary Table 1

### Source of mice

C57BL/6, *Gsdme*^*-/-*^ and *Nlrp3*^*-/-*^ mice were purchased from the Jackson Laboratories. *Gsdmd*^*-/-*^ and *Nlrc4*^*-/-*^ mice were a kind gift from Dr. Russell Vance (University of California, Berkeley). *Gsdmd*^*-/-*^*/Gsdme*^*-/-*^ mice were generated in-house. All transgenic mice were on a C57BL/6 background. As mice were bred in-house, we used approximately equal numbers of males and females, and ordered male and female mice from Jackson Laboratories. Animals were housed in pathogen free conditions in microisolator cages and were treated according to institutional guidelines following approval by the University of California, Irvine IACUC. We routinely use male and female mice aged 6-10 weeks. Although we noted the sex of each mice, we did not note any gender differences using mice as a source of cells, or in infection studies.

### Bacteria

DMSO stocks of *P. aeruginosa* PAO1F and T3SS mutant strains generated by Dr. Arne Rietsch were grown overnight in modified high salt LB (HSLB) broth containing 200 mM NaCl, 10 mM MgCl2, 0.5 mm CaCl2, and 5 mM EGTA to induce T3SS expression^55^. For experiments, overnight cultures were diluted 1:100 in fresh HSLB broth and grown to early log phase, OD_600_ of 0.18-0.2. Bacteria were washed in PBS, resuspended at 3x10^8^ bacteria/mL, and 20uL containing 6x 10^6^ bacteria were added to 200,000 neutrophils (MOI of 30) for in vitro stimulations.

### Human blood neutrophils

Whole blood was collected from male and female volunteers ages 18 and 65 years as part of the healthy blood donor program that is run by the Institute for Clinical and Translational Science at UC Irvine. This program has been approved by Institutional Review Board of the University of California (Irvine, CA), following informed consent, and donors are de-identified. Given that we used blood from only 6 volunteers, sex was not considered in the study design. Neutrophils were isolated by dextran sedimentation followed by density gradient centrifugation (Ficoll-Paque Plus, GE Healthcare) at 500 × *g* for 30 min, followed by lysis of erythrocytes with RBC lysis buffer (eBiosciences). This protocol routinely yields >90% purity as assessed by flow cytometry using anti-human CD16 and CD66b Abs (eBiosciences).

### Isolation of murine bone marrow and peritoneal neutrophils

Bone marrow neutrophils were collected from the tibias and femurs of immunocompetent C57BL/6 or gene knockout mice. Peritoneal neutrophils were elicited by injecting mice IP with 1 mL of a 9% sterile casein solution for 16-20 h followed by a second injection of casein 3 h prior to collection. Following lavage of the peritoneum, cells were drawn into cold PBS. Neutrophils were enriched via negative selection magnetic beads using the manufacturer’s recommended protocols (Biolegend MojoSort or STEMCELL EasySep). This protocol routinely yields >95% neutrophils as determined by flow cytometry using antibodies to Ly6G and CD11b. Neutrophils were resuspended in RPMI (Gibco) at the specified concentrations for in vitro assays.

### Generation of bone marrow derived macrophages (BMDM)

To derive macrophages, total bone marrow cells were collected by seeding total bone marrow cells into untreated culture flasks (Gen Clone, 25-214) containing 10 mL Dulbecco’s modified Eagle’s medium (DMEM; Gibco), 10% fetal bovine serum (FBS; Corning), and 10 ng/mL M-CSF (R&D Systems). On Day 2 and Day 4, non-adherent cells were removed by washing, and growth medium was replaced. On Day 6, BMDMs were detached using Cellstripper^TM^ (Corning, NY) following manufacturer’s directions and plated in media with 20 ng/mL GM-CSF (STEMCELL) for in vitro assays the following day.

### Cell stimulation conditions

Isolated neutrophils and macrophages were stimulated with ultrapure lipopolysaccharide (LPS) (Invivogen) at 500 ng/mL for 3 h, and 3 mM ATP (Sigma-Aldrich) was added for 1 h to selectively activate NLRP3. In some experiments, 20 ng/mL GM-CSF was also included in the cultures, which may affect P2X7 functional expression. To induce caspase-3 and GSDME activation, 100 ng/mL recombinant TNFα (R&D Systems) with 2 µM Birinapant Smac mimetic (MedChemExpress) or 2.5 ng/mL cycloheximide (final concentrations) were added for 4 h. The pan caspase inhibitor Z-VAD-FMK (APExBIO), the NLRP3 inhibitor MCC950 (Invivogen) and the ROS inhibitor diphenyleneiodonium chloride (DPI, Sigma) were dissolved in dimethyl sulfoxide (DMSO) (Thermo Fisher Scientific). Cells were pretreated with ZVAD at 50 µM for 30 min, with MCC920 at 2 µM for 40 min, with DPI at 10 µM for 20 min, or with the equivalent volume of DMSO as vehicle control. Cells were incubated with *Pseudomonas aeruginosa* strains at an MOI = 30 for 25 min for western blots or 1 h for cytokine production and LDH release. Neutrophils were suspended in media containing 20 ng/mL GM-CSF to reduce spontaneous activation of caspase-3. Macrophages were pretreated with 5 mM glycine for 30 min to inhibit macrophage pyroptosis as described^25,26^.

### Cytokine assays

Neutrophils (2x10^5^ /well) or macrophages (3.5x10^4^ /well) were stimulated as indicated. Cell-free supernatants were collected by centrifugation in V-bottom 96-well plates (PlateOne). Proteins were measured by ELISA following manufacturer’s recommendations (DuoSet, R&D).

IL-1β bioactivity was quantified using the HEK-blue IL-1R1 reporter cell-line (InvivoGen). 2.8 x10^5^ cells/mL in DMEM (GIBCO) complete media with or without anti- IL-1β neutralizing antibodies (R&D Systems) and incubated overnight at 37 C with 5%CO2. Supernatants were incubated with QUANTI-Blue (InvivoGen) for 30 minutes, and cytokines were measured on the Biotek Cytation-5 reader. IL-1 concentrations were calculated as pg/ml based on standards.

### Lactate dehydrogenase (LDH) assay for cell death

Cell cytotoxicity was determined by quantifying LDH release with CytoTox 96® Non-Radioactive Cytotoxicity Assay (Promega) according to the manufacturer’s instructions. Percentage cytotoxicity was calculated based on maximum LDH release following incubation with 1% Triton X-100.

### Western Blots

Cells (2-4x10^6^ BMNs or 2x10^6^ BMDMs) were lysed in 1x lysis buffer (CST). For neutrophils, we included 5 mM diisopropylfluorophosphate (DFP), which is a covalent inhibitor of neutral serine proteases such as neutrophil elastase. We found that DFP inhibits post-lysis processing of neutrophil proteins, including GSDMD^7^. Protein in 1-1.5 mL of supernatants was precipitated using 10% sodium deoxycholate and 100% TCA. The resulting pellets were washed with 100% acetone and solubilized in 0.2 M NaOH. BCA assays were run to determine protein concentrations in cell lysates. Unless otherwise noted, 10-30 μg total protein combined with 1-1.5 mL of TCA-precipitated supernatant were separated on 4-20% acrylamide gels via SDS-PAGE. Protein was transferred to PVDF membranes (Millipore) using the TransBlot Turbo semi-dry apparatus (Bio-Rad).

Antibodies for western blot were diluted as follows: anti-IL-1β (1:800), anti- β -actin (1:500), anti-GSDMD (1:500), anti-GSDME (1:1000), anti-caspase-3 (1:1000), HRP-conjugated secondary antibodies (1:1000). Membranes were developed using Supersignal West Femto Maximum Sensitivity Substrate (Thermo Scientific). Uncropped western blots are provided in Figs. S5-S10.

### Reactive oxygen species (ROS) quantification

Neutrophils were incubated with 50 μM luminol (Sigma) at 1x10^6^ cells/mL. Cells were plated in black-wall 96-well plates with an optically clear bottom (CoStar 3720) at 2x10^5^ neutrophils per well and incubated for 20-30 minutes. *P. aeruginosa* strains grown to log phase or other stimuli were added at indicated concentrations. Cells were incubated in a Cytation5 (BioTek) at 37 °C and luminescence was measure from each well every 2 min for 90 min. Area under the curve was calculated using Prism (Graphpad).

### Neutrophil extracellular traps

*Sytox*^*TM*^ *kinetic assay for extracellular DNA —* Enriched neutrophils were resuspended at 1x10^6^ cells/mL in RPMI with 20 ng/mL GM-CSF (R&D or STEMCELL) (for murine neutrophils) or RPMI with 2% FBS (for human neutrophils) with 1 μM Sytox Green (Invitrogen) and plated in 96-well plates at 200,000 neutrophils per well. Log phase *P. aeruginosa* and other stimuli were added at indicated concentrations, and fluorescence (485 Ex/525 Em) was quantified every 15 min for 16 h using a Cytation5 (BioTek) at 37 °C with 5% CO2. The area under the curve between time 0- 6 h was calculated using Prism (Graphpad).

*Histone citrullination —* Enriched casein-elicited peritoneal mouse neutrophils were resuspended in RPMI with 20 ng/mL GM-CSF (R&D or STEMCELL) at 1x10^6^ cells/mL. 500,000 neutrophils were seeded onto sterile 12 mm circular glass coverslips (#1.5, Thermo or EMS) in 24-well plates and stimulated as indicated. Coverslips were washed gently with cold PBS and fixed for 20 minutes at 4 °C in 2% PFA in PBS. For washes and fixation, CaCl_2_ and MgCl_2_ were added to PBS at 0.9 mM and 0.5 mM, respectively. Coverslips were incubated for 1 h in 10% normal donkey serum in PBS with 0.1% Triton X-100 and incubated overnight with indicated primary antibodies in blocking buffer. Coverslips were then washed in PBS, incubated in secondary antibodies for 2 h, and counterstained in 0.1 μg/mL DAPI before being mounted to slides using VectaSHIELD HardSet (Vector Labs) and sealed with nail polish. Neutrophils were imaged using an LSM700 confocal microscope (Zeiss) and processed with FIJI/ImageJ (NIH) or Zen 2 blue edition (Zeiss).

### Murine model of *P. aeruginosa* corneal infection

Overnight cultures of *P. aeruginosa* PAO1F or mutant strains were grown to log phase (OD_600_ of 0.2) in LB broth, then washed and resuspended in PBS. C57BL/6 and transgenic mice 6–10 week old were anesthetized with ketamine/xylazine solution, the corneal epithelium was abraded with three parallel scratches using a sterile 26-gauge needle, and 2 μL of a suspension of bacteria were added topically (approximately 5×10^4^ bacteria per eye).

After 24 h or 48 h, mice were euthanized and corneas were imaged by brightfield to detect opacification, or by fluorescence microscopy to detect GFP-expressing bacteria. Fluorescent intensity images were quantified using Image J software (NIH). For live bacteria, whole eyes were homogenized in PBS using a TissueLyser II (Qiagen, 30 Hz for 3 minutes), and homogenates were serially diluted and streaked on LB plates for quantification of colony forming units (CFU) by manual counting. CFU were also determined at 2 h to confirm the inoculum. For western blot and cytokine analyses, corneas were carefully dissected to remove vascularized iris tissue, placed in 1x lysis buffer (CST) containing 5 mM DFP, and processed as described above.

### Flow cytometry

Corneas from infected mice were dissected and incubated with 3 mg/ml collagenase (C0130; Sigma-Aldrich) in RPMI (Life Technologies), with 1% HEPES (Life Technologies), 1% penicillin-streptomycin (Life Technologies), and 0.5% BSA (Fisher Bioreagents) for 1 h and 15 min at 37◦C. All subsequent steps were at 4^0^C. Cells were recovered following centrifugation and incubated 5 min with anti-mouse CD16/32 Ab (BioLegend) to block Fc receptors, then 20 min with anti-mouse CD45-allophycocyanin, Ly6GBV510, Ly6C-PE-Cy7, CD11b-PETxRed, CCR2-BV421, or F4/80-FITC (BioLegend) and fixable viability dye (BD Biosciences). Cells were washed with FACS buffer and quantified using an ACEA Novocyte flow cytometer and NovoExpress software. The gating strategy identified total cells in infected corneas by forward and side scatter, followed by gating on single cells and live cells were identified using the E780 viability dye (Biolegend). Neutrophils were identified as CD45 + , CD11b + , Ly6G + , CCR2-, and monocytes were CD45 + , CD11b + , Ly6G- CCR2 + . The gating strategy is shown in Fig. S3K.

### Statistics and Reproducibility

All experiments were repeated at least 3 times, and statistics were based on biological replicates. Corneal infection studies utilized at least 5 mice per group, which is sufficient to generate statistical significance was determined using one-way or two-way ANOVA with either HSD Tukey’s post hoc analysis or by 1-way ANOVA followed by Kruskal-Wallis post-test for in vivo studies. In experiments with only two variables, we used unpaired Students t-test. Outliers were removed using the ROUT method (Q = 1%). All analyses were performed using GraphPad Prism. Differences were considered significant when the *P* value was <0.05. Experiments were not randomized; and investigators were not blinded to allocation during experiments and outcome assessment.

### Reporting summary

Further information on research design is available in the Nature Portfolio Reporting Summary linked to this article.

### Supplementary information

Supplementary Information

Reporting Summary

### Source data

Source Data

## Data Availability

The authors declare that all data underlying the findings of the current study are available within the article and Supplementary Information or available from the corresponding authors upon request. Source data are provided with this paper.
