# Supplementary material for: NLRP3 selectively drives IL-1β secretion by Pseudomonas aeruginosa infected neutrophils and regulates corneal disease severity
Source: Nat Commun. 2023 Sep 20;14:5832. doi: 10.1038/s41467-023-41391-7 (PMC10511713; doi:10.1038/s41467-023-41391-7)
Supplement: Supplementary file 1 — Supplementary Information [file 41467_2023_41391_MOESM1_ESM.pdf]

REAGENT or RESOURCE	SOURCE	IDENTIFIER
**Antibodies**		
Goat Anti-Mouse Il-1 beta / il-1f2 Polyclonal antibody, Unconjugated	R&D Systems	RRID:AB_416684 Cat# AF-401-NA
Mouse anti β-actin (C-4)	Santa Cruz	RRID:AB_2714189 Cat# sc-47778
Anti-DFNA5/GSDME antibody [EPR19859]	Abcam	RRID:AB_2737000 Cat# ab215191
Anti-GSDMD antibody [EPR19828]	Abcam	RRID:AB_2783550 Cat# ab209845
Rabbit anti-caspase-3	Cell Signaling	RRID:AB_331439 Cat# 9662
Goat anti-rabbit IgG, HRP-linked	Cell Signaling	RRID:AB_2099233 Cat# 7074
Horse anti-mouse IgG, HRP-linked	Cell Signaling	RRID:AB_330924 Cat# 7076
Rabbit anti-goat IgG, HRP-linked	R&D Systems	Cat# HAF017
Rabbit anti-histone H3 (citrulline R2 + R8 + R17)	abcam	RRID:AB_304752 Cat# ab5103
Goat anti-Rat IgG (H+L) Cross-Adsorbed Secondary Antibody, Alexa Fluor™ 647	Invitrogen	RRID:AB_141778 Cat# A-21247
Rabbit anti-Mouse IgG (H+L) Cross-Adsorbed Secondary Antibody, Alexa Fluor™ 488	Invitrogen	RRID:AB_2534106 Cat# A-11059
Rabbit anti-Mouse IgG (H+L) Cross-Adsorbed Secondary Antibody, Alexa Fluor™ 555	Invitrogen	RRID:AB_2535848 Cat# A-21427
Brilliant Violet 510 anti-mouse Ly-6G Antibody, clone 1A8	Abcam	Cat# ab222736
AmCyan anti-mouse Ly-6G Antibody		
Fixable viability dye, e780	BioLegend	Cat#503203
PE anti-mouse/human CD11b Antibody, clone M1/70	BioLegend	RRID:AB_1186099; Cat#127608
PE/Dazzle™ 594 anti-mouse/human CD11b Antibody	BioLegend	RRID:AB_1574975 Cat# 101320
**Chemicals, peptides, and recombinant proteins**		
LPS-EK ULTRAPURE (Ultrapure lipopolysaccharide from *E. coli* K12)	InvivoGen	Cat# tlrl-peklps
Adenosine 5′-triphosphate disodium salt hydrate (ATP)	Sigma Aldrich	Ca# A6419-1G
Recombinant murine M-CSF (rmM-CSF)	STEMCELL	Cat# 78059.1
Recombinant murine GM-CSF (rmGM-CSF)	STEMCELL	Cat# 78017
Recombinant human TNFa (rhTNFa)	R&D Systems	Cat# 210-TA
Birinapant (SMAC mimetic)	MedChemExpress	Cat# HY-16591/CS-1719
MCC950 (NLRP3 inhibitor)	Invivogen	inh-mcc
Diphenyleneiodonium chloride (DPI)	Sigma Aldrich	Cat# D2926
SYTOX Green	Invitrogen	Cat# S 7020
Phorbol myristate acetate (PMA)	Sigma Aldrich	Cat# P1585
Z-VAD-FMK (pan-caspase inhibitor)	APExBIO	Cat# A1902
Ac-YVAD-cmk (caspase-1 inhibitor)	Sigma Aldrich	Cat# SML0429-5MG
Z-IETD-FMK (caspase 8 inhibitor)	APExBIO	Cat# B3232
Q-VD(OMe)-OPh (pan-caspase inhibitor)	APExBIO	Cat# A8165

Z-DEVD-FMK (caspase 3 inhibitor)	APExBIO	Cat# A1920
Necrosulfonamide (NSA)	TOCRIS bioscience	1360614-48-7
Disulfiram (GSDMD inhibitor)	MedChemExpress	Cat# HY-B0240/CS-2209
LDC7559	MedChemExpress	HY-111674/CS-0089815
Diisopropylfluorophosphate (DFP; protease inhibitor)	Sigma Aldrich	Cat# D0879
Casein sodium salt from bovine milk	Sigma Aldrich	Cat# C8654-500G
Collagenase from *Clostridium histolyticum*	Sigma Aldrich	CAS# 54724-00-4<br>Cat# C7821-5G
Cell Lysis Buffer (10X)	Cell Signaling	Cat# 9803
Luminol	Sigma Aldrich	Cat# 123072-5G
**Commercial assays**		
EasySep™ Mouse Neutrophil Enrichment kit	STEMCELL	Cat# 19762A
Mojosort Mouse Neutrophil Isolation Kit	BioLegend	Cat# 480058
CytoTox 96 (LDH release assay)	Promega	G1780
DuoSet mouse IL-1b ELISA	R&D Systems	DY401-05
BD Cytofix/Cytoperm Fixation/Permeablization Kit	BD Biosciences	Cat#554714<br>RRID:AB_2869008
Cell line: HEK-Blue™ IL-1R Cells (HEK293 reporter cells for human and murine IL-1α & IL-1β cytokines)	InvivoGen	Cat# hkb-il1r
**Mouse lines:**		
Mouse: C57BL/6J	The Jackson Laboratory (JAX)	Cat# 000664<br>RRID:IMSR_JAX:000664
Mouse: *gsdme*$^{-/-}$	The Jackson Laboratory (JAX)	Cat# 032411<br>RRID:IMSR_JAX:032411
Mouse: *gsdmd*$^{-/-}$	Dr. Russell Vance	
Mouse: *gsdmd*$^{-/-}$ *gsdme*$^{-/-}$	Bred in house	
Mouse: Caspase 1-/-	The Jackson Laboratory (JAX)	Cat# 032662<br>RRID:IMSR_JAX:032662
Mouse: *Caspase 1/11-/-*	The Jackson Laboratory (JAX)	Cat# 016621<br>RRID:IMSR_JAX:016621
Mouse: *elane-/- (neutrophil elastase)*	Dr. Timothy Kern, University of California, Irvine	
**Software**		
Excel	Microsoft	RRID:SCR_016137
Image Lab (for use with Bio-Rad ChemiDoc)	BioRad	RRID:SCR_014210
Photoshop	Adobe	RRID:SCR_014199
Illustrator	Adobe	RRID:SCR_010279
Biorender	Biorender	RRID:SCR_018361
Zen blue microscope imaging	Zeiss	RRID:SCR_013672
BioTek Gen5	Agilent	RRID:SCR_017317
GraphPad Prism	GraphPad	RRID:SCR_002798
NovoExpress Software	Agilent	

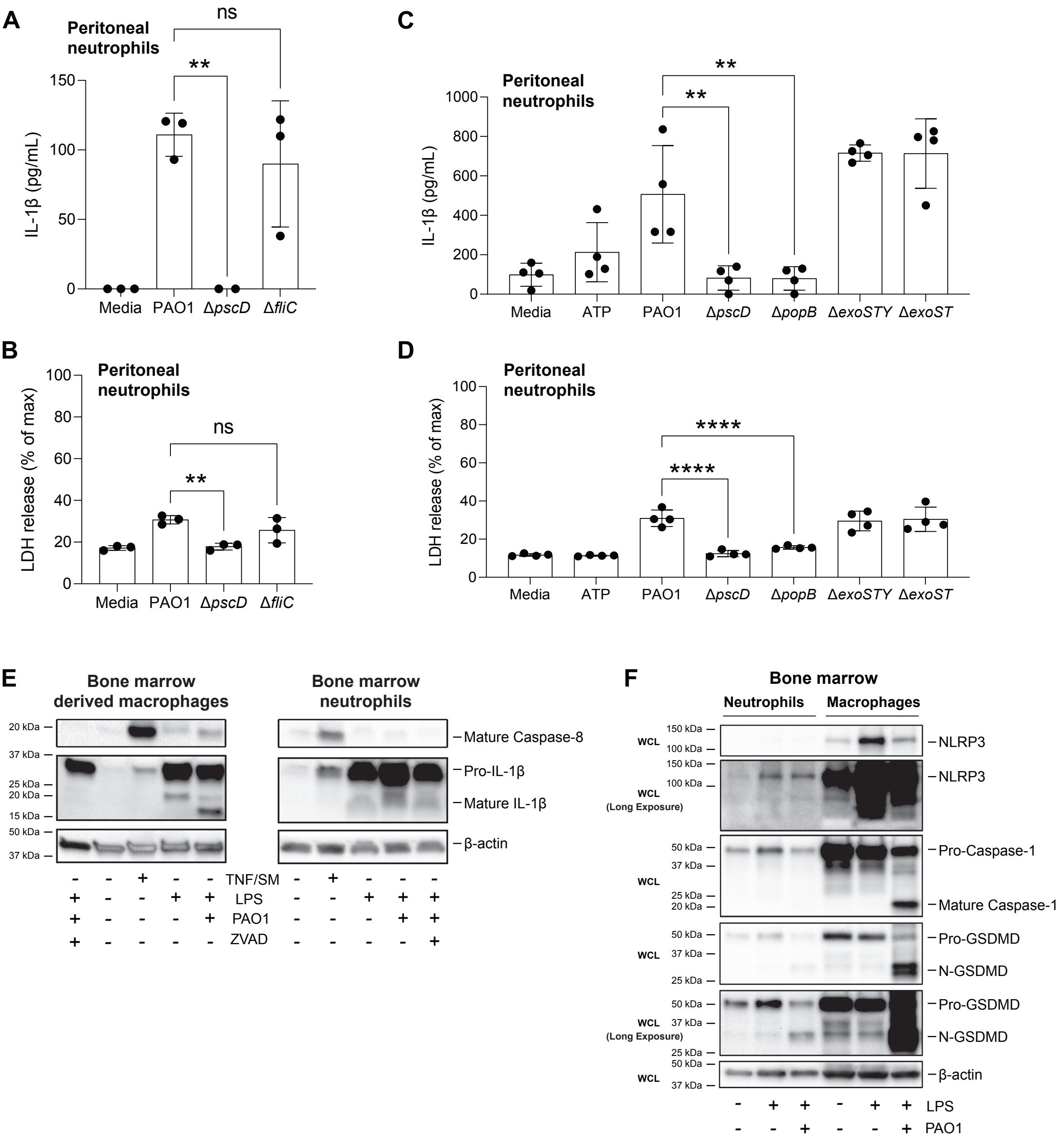

Figure S1. IL-1β secretion by peritoneal neutrophils, Caspase-8 and IL-1β cleavage, and relative production of inflammasome proteins.

A-D. IL-1β secretion and pyroptosis in peritoneal neutrophils. Neutrophils were isolated from the peritoneal cavity and purified by negative bead selection following intraperitoneal injection of casein to induce sterile inflammation (-24h, -3h i.p. injections). **A,C.** IL-1β secretion; **B,D.** LDH release. Peritoneal neutrophils were infected for 1h with PAO1 or with T3SS or flagellin (Δ*fliC)* mutants. *Peritoneal neutrophils were not primed with LPS.* Data points represent biological replicates from 2-3 repeat experiments.

E. Caspase-8 and IL-1β cleavage in bone marrow derived macrophages and bone marrow neutrophils. Cells were stimulated with TNF-α and SMAC mimetic (TNF/SM) or LPS/PAO1 in the presence of the pan-caspase inhibitor ZVAD, and whole cell lysates with supernatants were prepared for western blot analysis for caspase-8 and IL-1β.

F. Relative production of inflammasome proteins by bone marrow neutrophils and BM derived macrophages. 1x10^{6} neutrophils and macrophages were either left unstimulated or incubated for 3h with LPS before being infected for 1h with PAO1. Whole cell lysates were processed for western blots to examine the relative production of NLRP3, caspase-1 and GSDMD.

Western blots are representative of 3 repeat experiments. Each data point represents one independent experiment (2-4 biological replicates); error bars are mean+/- standard deviation (SD). **A-D**: One way ANOVA with Tukey's post-hoc analysis (Mean +/- SD). **** represents p<0.0001, ** is <0.01. ns: not statistically significant.

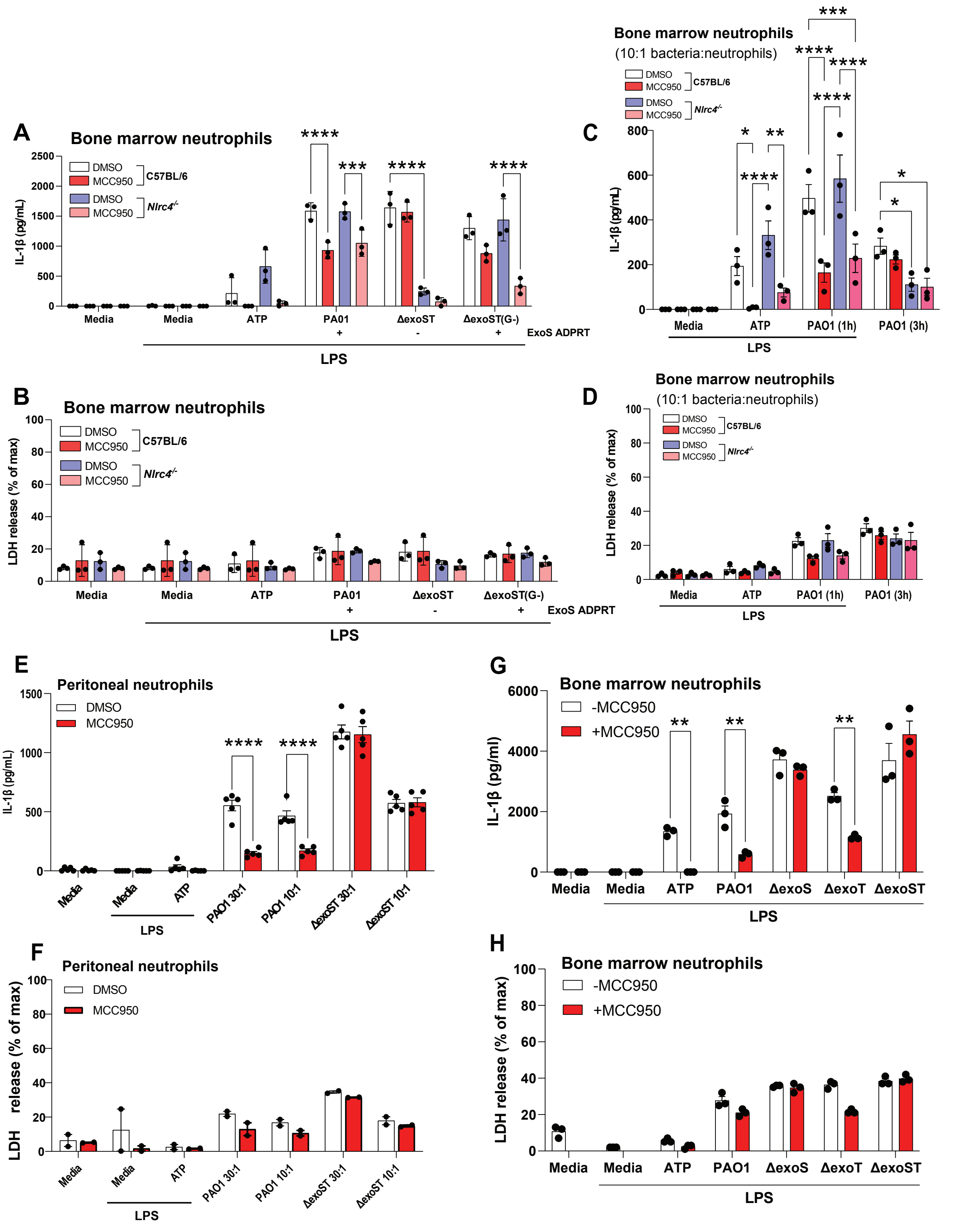
A
Bone marrow neutrophils
IL-1β (pg/mL)
DMSO
MCC950
C57BL/6
Nlrc4-/-
Media
ATP
PA01
ΔexoST
ΔexoST(G-)
ExoS ADPRT
LPS
B
LDH release (% of max)
C
(10:1 bacteria:neutrophils)
PAO1 (1h)
PAO1 (3h)
D
E
Peritoneal neutrophils
PAO1 30:1
PAO1 10:1
ΔexoST 30:1
ΔexoST 10:1
F
G
-MCC950
+MCC950
IL-1β (pg/ml)
PAO1
ΔexoS
ΔexoT
ΔexoST
H

Figure S2. MCC950 inhibition of IL-1β secretion by C57BL/6 and *Nlrc4$^{-/-}$* bone marrow neutrophils and peritoneal neutrophils.

A, B. Neutrophils isolated from C57BL/6 and *Nlrc4$^{-/-}$* mice were LPS primed for 3h and infected with PAO1 or mutants for 1h in the presence of MCC950 or DMSO at a multiplicity of infection (MOI) of 30:1 (bacteria: neutrophils). IL-1β and LDH in culture supernatants were quantified. **C, D.** IL-1β and LDH in C57BL/6 and *Nlrc4$^{-/-}$* mouse neutrophils that were either LPS primed and infected with PAO1 at an MOI of 10:1, or not primed and infected with PAO1 for 3h as described by Santoni *et. Al.*, *PLOS Pathogens 2022*. **E, F.** IL-1β and LDH release by peritoneal neutrophils from C57BL/6 mice were not LPS primed and were infected with 30:1 or 10:1 MOI for 1h in the presence of MCC950. **G, H.** IL-1β and LDH in bone marrow neutrophils from C57BL/6 mice primed and stimulated as in **A, B**. Two-way ANOVA with Tukey's post-hoc analysis (Mean +/- SD). Each data point represents one independent experiment (2-5 biological replicates); error bars are mean+/- SEM. A-D: **** represents p<0.0001, *** is p<0.001; ** is <0.01, * is <0.05.

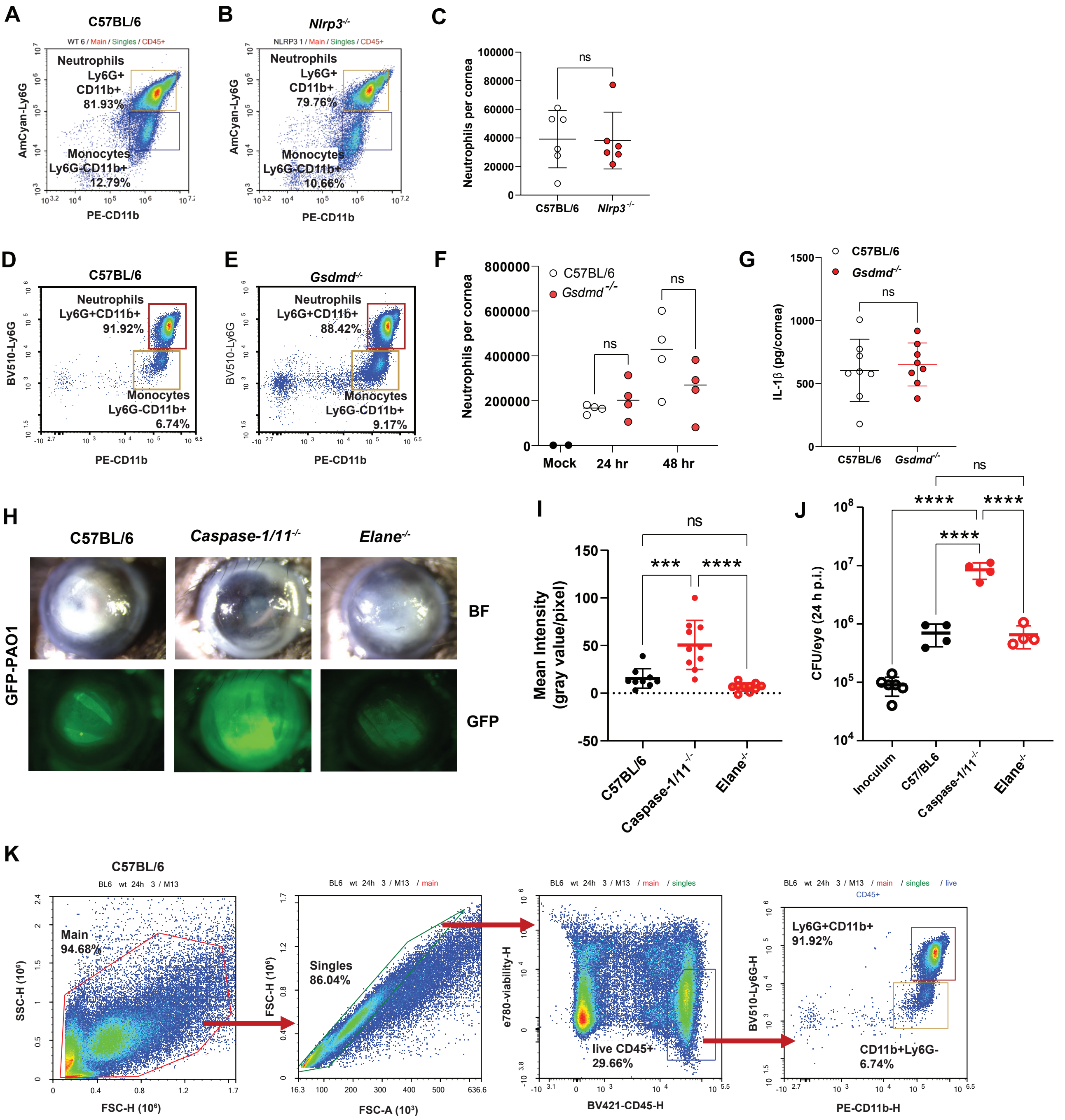
A
C57BL/6
WT 6 / Main / Singles / CD45+
Neutrophils Ly6G+ CD11b+ 81.93%
Monocytes Ly6G-CD11b+ 12.79%
AmCyan-Ly6G
PE-CD11b
B
Nlrp3-/-
NLRP3 1 / Main / Singles / CD45+
Neutrophils Ly6G+ CD11b+ 79.76%
Monocytes Ly6G-CD11b+ 10.66%
AmCyan-Ly6G
PE-CD11b
C
Neutrophils per cornea
ns
C57BL/6
Nlrp3-/-
D
C57BL/6
Neutrophils Ly6G+CD11b+ 91.92%
Monocytes Ly6G-CD11b+ 6.74%
BV510-Ly6G
PE-CD11b
E
Gsdmd-/-
Neutrophils Ly6G+CD11b+ 88.42%
Monocytes Ly6G-CD11b+ 9.17%
BV510-Ly6G
PE-CD11b
F
Neutrophils per cornea
C57BL/6
Gsdmd-/-
ns
Mock
24 hr
48 hr
G
IL-1β (pg/cornea)
C57BL/6
Gsdmd-/-
ns
H
C57BL/6
Caspase-1/11-/-
Elane-/-
GFP-PAO1
BF
GFP
I
Mean Intensity (gray value/pixel)
ns
***
****
C57BL/6
Caspase-1/11-/-
Elane-/-
J
CFU/eye (24 h p.i.)
ns
****
****
****
Inoculum
C57/BL6
Caspase-1/11-/-
Elane-/-
K
C57BL/6
BL6 wt 24h 3 / M13
Main 94.68%
SSC-H (10⁶)
FSC-H (10⁶)
BL6 wt 24h 3 / M13 / main
Singles 86.04%
FSC-H (10⁶)
FSC-A (10³)
BL6 wt 24h 3 / M13 / main / singles
live CD45+ 29.66%
e780-viability-H
BV421-CD45-H
BL6 wt 24h 3 / M13 / main / singles / live CD45+
Ly6G+CD11b+ 91.92%
CD11b+Ly6G- 6.74%
BV510-Ly6G-H
PE-CD11b-H

Figure S3. Neutrophil infiltration in PAO1 infected corneas, neutrophil elastase (*Elane*)-/- mice, and gating strategy.

A-F. Neutrophil infiltration in PAO1 infected corneas. A-C. Neutrophils and monocytes from C57BL/6 and *Nlrp3*$^{-/-}$ corneas 24h after infection with PAO1. Cells were recovered from infected corneas following collagenase digestion and processed for flow cytometry. Neutrophils were defined as CD45^{+}, Ly6G^{+}, CD11b^{+}, Ly6C^{-}; monocytes were defined as CD45^{+}, Ly6G^{-}, CD11b^{+}, Ly6C^{+}. **D-G.** Flow cytometry of neutrophils and monocytes from C57BL/6 and *Gsdmd*$^{-/-}$ corneas 24h post-infection with PAO1. **A,B, D,E.** representative flow cytometry scatter plots; **C,F.** quantification of total neutrophils.

G. IL-1β in homogenates from infected corneas measured by ELISA.

H-J. Corneal infection in Caspase-1/11 and neutrophil elastase (*Elane*) gene knockout mice. H. Representative images of corneas of C57BL/6, *caspase-1,11*$^{-/-}$, and *Elane*$^{-/-}$ mice infected with PAO1 expressing green fluorescent protein (GFP). GFP expression **(I)** and CFU (**J**) showing significant difference between C57BL/6 and *caspase-1,11*$^{-/-}$, but not between C57BL/6 and *Elane*$^{-/-}$ mice. Data points are biological replicates. Genotyping confirmed that these were *Elane*$^{-/-}$ mice (not shown).

K. Gating strategy for A-F. Side scatter (SSC) and forward scatter (FSC) of collagenase digested, PAO1 infected C57BL/6 corneas. Gated for singlets and live CD45^{+} cells, which were stained for Ly6G^{+} CD11b^{+} neutrophils.

For all graphs, each data point represents one independent experiment (2-5 biological replicates). **C, G**: Student's 2-tailed t-tests. **F, I,J**. One-way ANOVA with Tukey's post-hoc analysis. Error bars are mean+/- SD. **** represents p<0.0001, *** is p<0.001; ** is <0.01, * is <0.05. ns: not statistically significant.

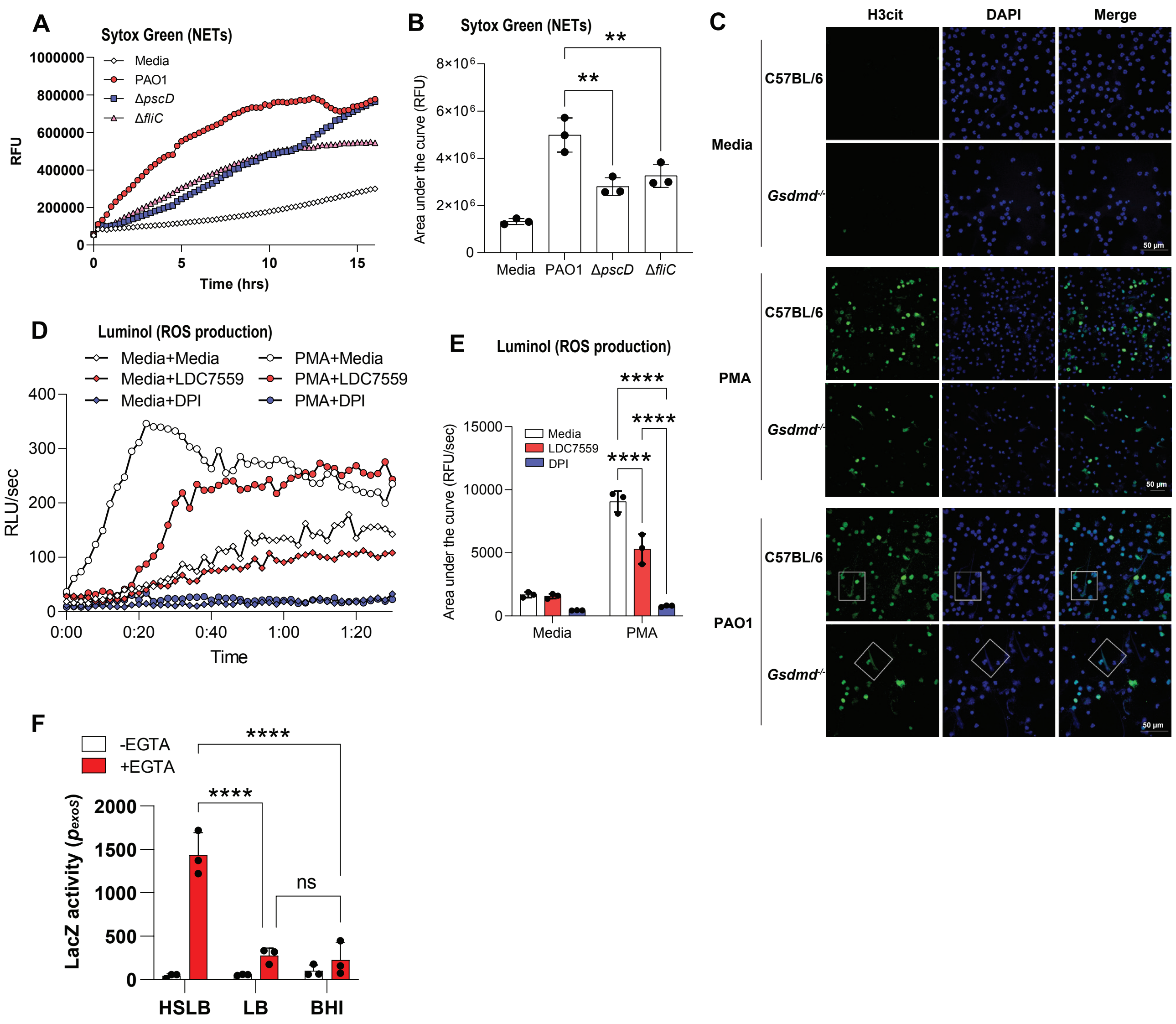

Figure S4. Neutrophil extracellular trap formation induced by Δ*pscD* and Δ*fliC* mutants, LDC7559 inhibition of ROS, and ExoS expression in HSLB media.

A, B. Representative time course and combined data of NETosis induced by PAO1 compared with Δ*pscD* and Δ*fliC* mutants (measured by Sytox^{TM} green release over 16h). **C.** Representative images of H3Cit in PMA and PAO1 infected peritoneal neutrophils. **D,E.** Inhibition of ROS production by PMA–stimulated neutrophils incubated with DPI or LDC7559, which is consistent with data from Dixit *et al. Cell, 2021.* Representative time course (**D**) and combined biological replicates (**E**). **F.** Induction of *P. aeruginosa* T3SS gene expression was monitored using a *lacZ* reporter gene inserted at the *exoS* locus on the chromosome (strain RP1868, PAO1F Δ*exoS*:GFP-*lacZ*). Bacteria were grown in the indicated medium: high salt LB (HSLB), standard LB with 5g/L NaCl, or brain heart infusion (BHI) broth. Secretion (and T3SS gene expression) were up-regulated by adding EGTA to chelate Ca^{++}, and LacZ activity was assayed as previously described. For all graphs, each data point represents one independent experiment (3 biological replicates). **B**: One-way ANOVA with Tukey's post-hoc analysis. **E,F**: 2-way ANOVA with Tukey's post-hoc analysis. Error bars are mean+/- SD. **** represents p<0.0001; ** is <0.01, ns: not statistically significant.

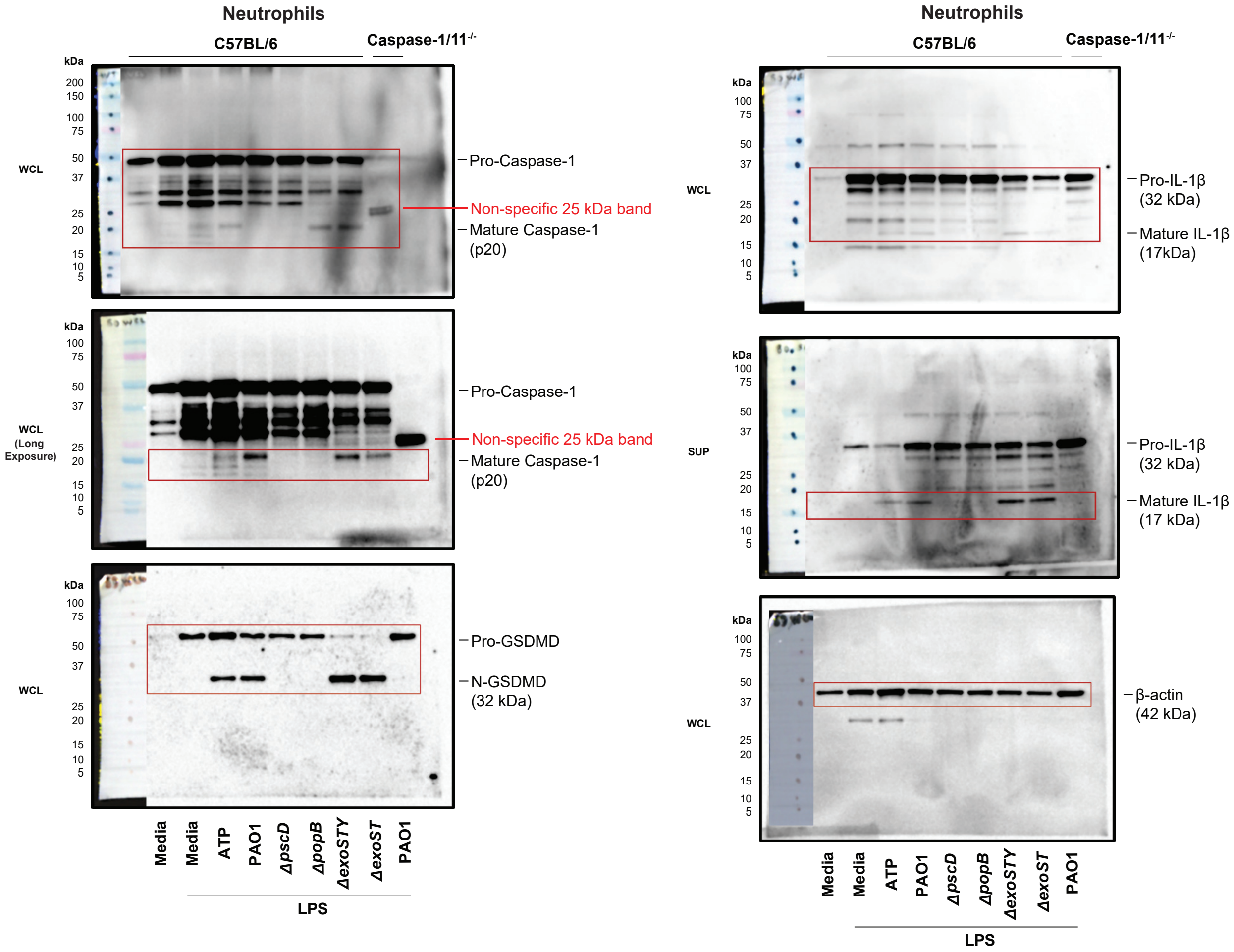

Figure S5. Un-cropped western blot images for Figure 1D.
Red boxes indicate where each image was cropped for the final figures.

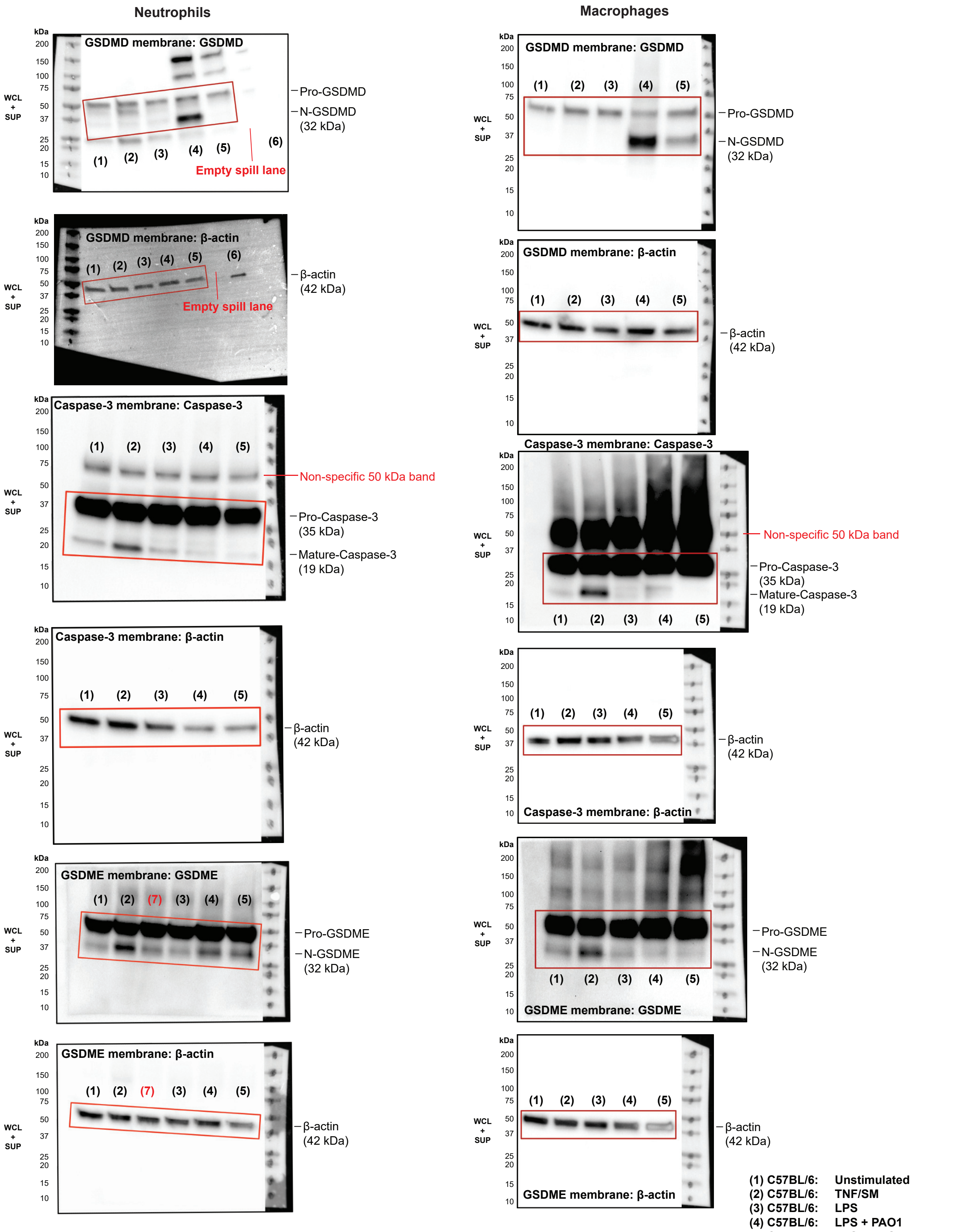

Figure S6. Un-cropped western blot images for Figure 1E and 1F.
Red boxes indicate where each image was cropped for the final figures.

(1) C57BL/6: Unstimulated
(2) C57BL/6: TNF/SM
(3) C57BL/6: LPS
(4) C57BL/6: LPS + PAO1
(5) C57BL/6: LPS + PAO1 + ZVAD
(6) GSDMD,E$^{-/-}$: PAO1
(7) C57BL/6: TNF/CHX

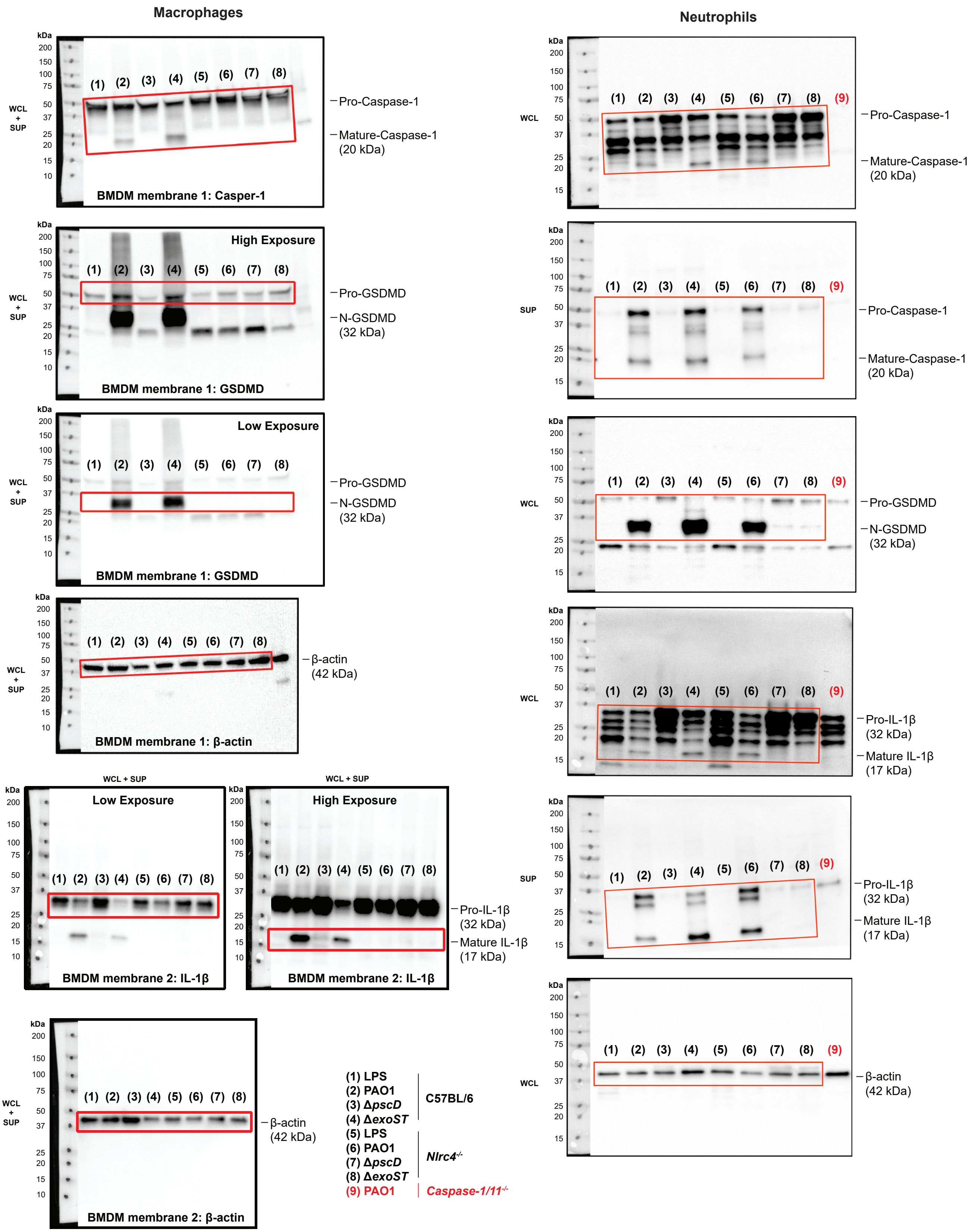

Figure S7. Un-cropped western blot images for Figure 2E.
Red boxes indicate where each image was cropped for the final figures.

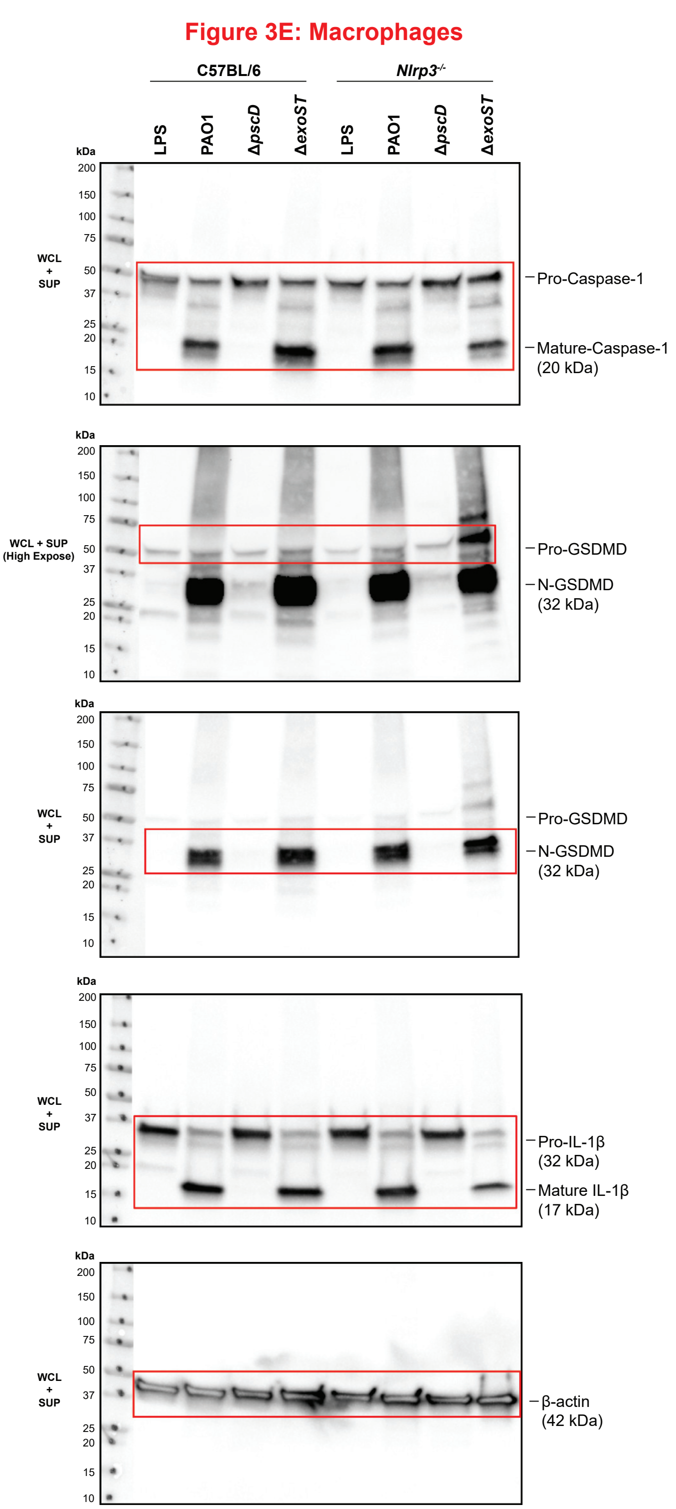

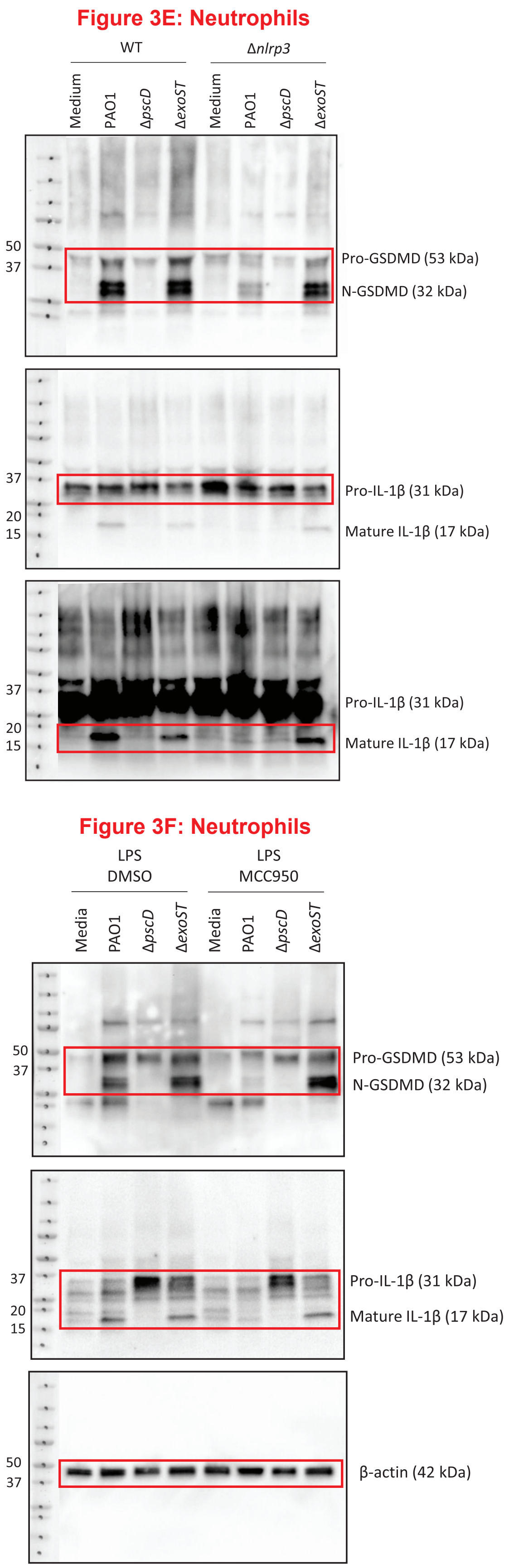

Figure S8. Un-cropped western blot images for Figure 3E and 3F.
Red boxes indicate where each image was cropped for the final figures.

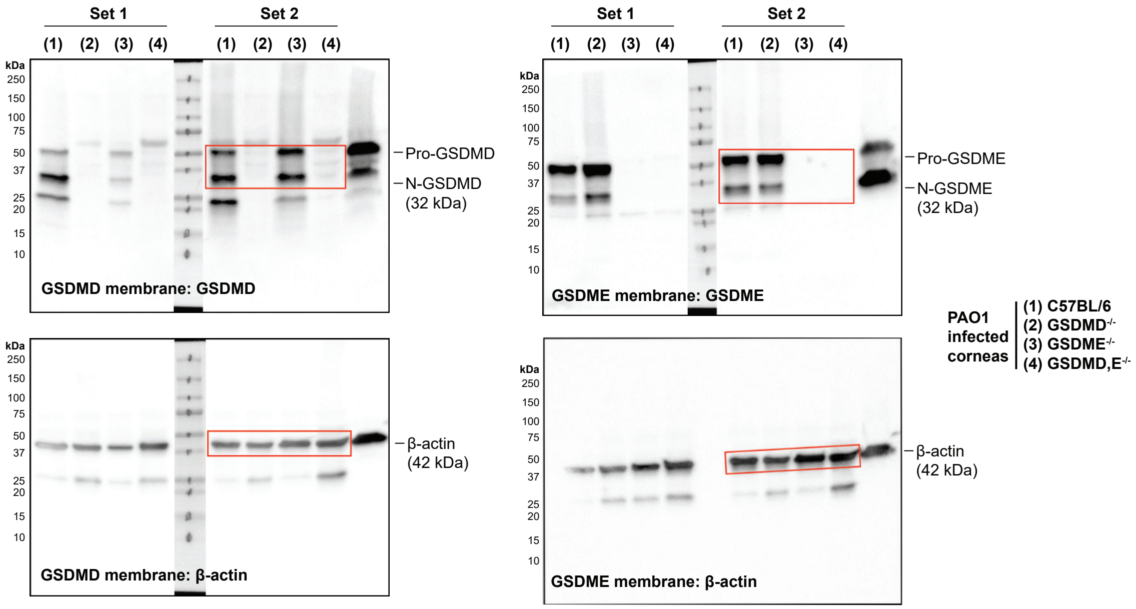

Figure S9. Un-cropped western blot images for Figure 4H.
Red boxes indicate where each image was cropped for the final figures. Set 1 and Set 2 are corneas from two different groups of mice.

Supplemental Figure 1E

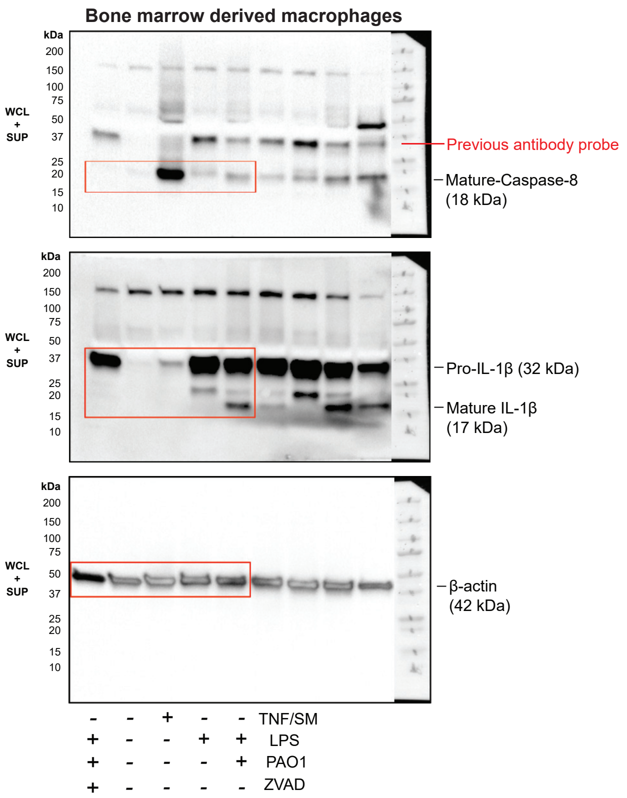

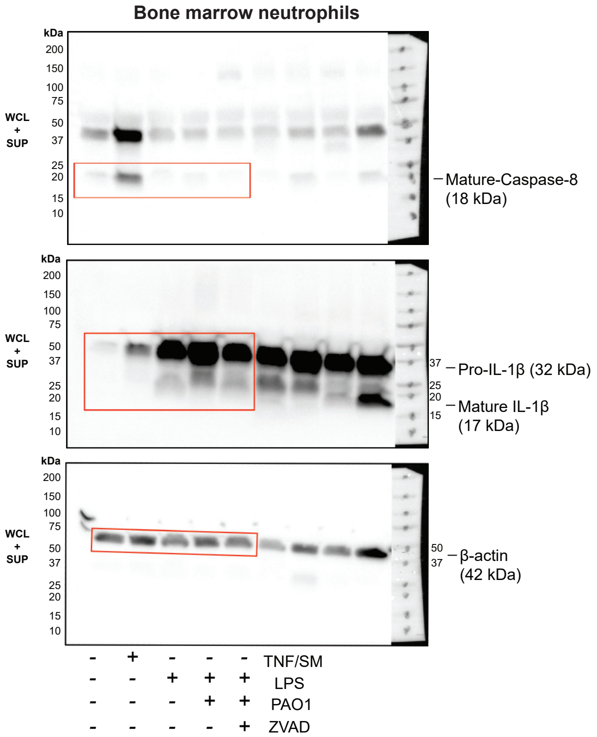

Supplemental Figure 1F

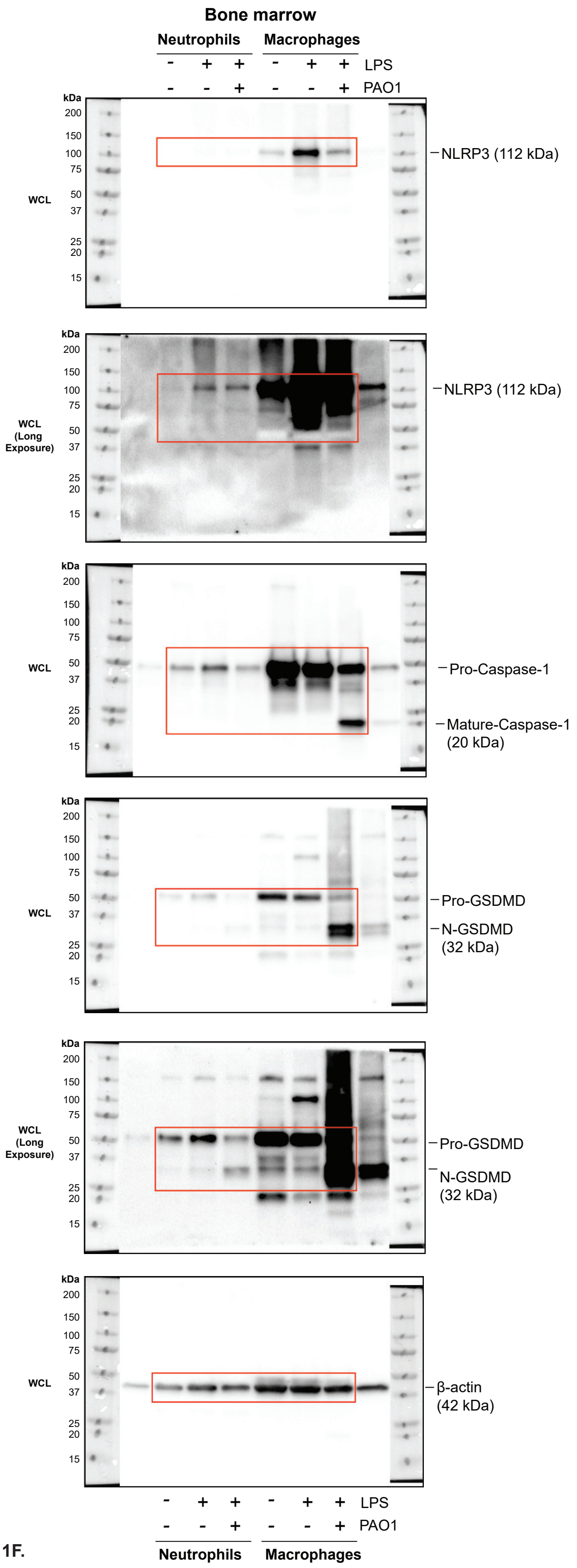

Figure S10. Un-cropped western blot images for Supplemental Figure 1E and 1F.
Red boxes indicate where each image was cropped for the final figures.
