# Supplementary material for: NLRP3 selectively drives IL-1β secretion by Pseudomonas aeruginosa infected neutrophils and regulates corneal disease severity
Source: Nat Commun. 2023 Sep 20;14:5832. doi: 10.1038/s41467-023-41391-7 (PMC10511713; doi:10.1038/s41467-023-41391-7)
Supplement: Supplementary file 2 — Reporting Summary [file 41467_2023_41391_MOESM2_ESM.pdf]

nature portfolio

Corresponding author(s): Eric Pearlman

Last updated by author(s): 6-14-2023

Reporting Summary

Nature Portfolio wishes to improve the reproducibility of the work that we publish. This form provides structure for consistency and transparency in reporting. For further information on Nature Portfolio policies, see our Editorial Policies and the Editorial Policy Checklist.

Statistics

For all statistical analyses, confirm that the following items are present in the figure legend, table legend, main text, or Methods section.

n/a	Confirmed	
☐	☒	The exact sample size (*n*) for each experimental group/condition, given as a discrete number and unit of measurement
☒	☐	A statement on whether measurements were taken from distinct samples or whether the same sample was measured repeatedly
☐	☒	The statistical test(s) used AND whether they are one- or two-sided<br>*Only common tests should be described solely by name; describe more complex techniques in the Methods section.*
☒	☐	A description of all covariates tested
☒	☐	A description of any assumptions or corrections, such as tests of normality and adjustment for multiple comparisons
☒	☐	A full description of the statistical parameters including central tendency (e.g. means) or other basic estimates (e.g. regression coefficient) AND variation (e.g. standard deviation) or associated estimates of uncertainty (e.g. confidence intervals)
☒	☐	For null hypothesis testing, the test statistic (e.g. *F*, *t*, *r*) with confidence intervals, effect sizes, degrees of freedom and *P* value noted<br>*Give P values as exact values whenever suitable.*
☒	☐	For Bayesian analysis, information on the choice of priors and Markov chain Monte Carlo settings
☒	☐	For hierarchical and complex designs, identification of the appropriate level for tests and full reporting of outcomes
☒	☐	Estimates of effect sizes (e.g. Cohen's *d*, Pearson's *r*), indicating how they were calculated

Our web collection on statistics for biologists contains articles on many of the points above.

Software and code

Policy information about availability of computer code

Data collection	N/A
Data analysis	N/A

For manuscripts utilizing custom algorithms or software that are central to the research but not yet described in published literature, software must be made available to editors and reviewers. We strongly encourage code deposition in a community repository (e.g. GitHub). See the Nature Portfolio guidelines for submitting code & software for further information.

Data

Policy information about availability of data

All manuscripts must include a data availability statement. This statement should provide the following information, where applicable:

- Accession codes, unique identifiers, or web links for publicly available datasets
- A description of any restrictions on data availability
- For clinical datasets or third party data, please ensure that the statement adheres to our policy

All data supporting the findings of this study are available within the paper and its Supplementary Information.

Research involving human participants, their data, or biological material

Policy information about studies with human participants or human data. See also policy information about sex, gender (identity/presentation), and sexual orientation and race, ethnicity and racism.

Reporting on sex and gender	N/A
Reporting on race, ethnicity, or other socially relevant groupings	N/A
Population characteristics	N/A
Recruitment	N/A
Ethics oversight	N/A

Note that full information on the approval of the study protocol must also be provided in the manuscript.

Field-specific reporting

Please select the one below that is the best fit for your research. If you are not sure, read the appropriate sections before making your selection.

- [x] Life sciences
- [ ] Behavioural & social sciences
- [ ] Ecological, evolutionary & environmental sciences

For a reference copy of the document with all sections, see nature.com/documents/nr-reporting-summary-flat.pdf

Life sciences study design

All studies must disclose on these points even when the disclosure is negative.

Sample size	Statistical significance was determined using unpaired t-test or two-way ANOVA with either HSD Tukey's post hoc analysis or by 1-way ANOVA followed by Kruskal-Wallis post-test (GraphPad Prism). Differences were considered significant when the P value was <0.05.
Data exclusions	For infection studies, outliers were removed with ROUT method (Q=1%) using GraphPad Prism software.
Replication	All experiments were repeated at least 3 times to generate biological replicates. Animal infection studies comprised a minimum of 5 mice/ group.
Randomization	There was no randomization - animals were separated into groups based on their genotypes and/or treatment
Blinding	Blinding was not required for these studies because they were distinct genomic or treatment groups.

Reporting for specific materials, systems and methods

We require information from authors about some types of materials, experimental systems and methods used in many studies. Here, indicate whether each material, system or method listed is relevant to your study. If you are not sure if a list item applies to your research, read the appropriate section before selecting a response.

Materials & experimental systems

n/a	Involved in the study	
[ ]	[x]	Antibodies
[x]	[ ]	Eukaryotic cell lines
[x]	[ ]	Palaeontology and archaeology
[ ]	[x]	Animals and other organisms
[x]	[ ]	Clinical data
[x]	[ ]	Dual use research of concern
[x]	[ ]	Plants

Methods

n/a	Involved in the study	
[x]	[ ]	ChIP-seq
[ ]	[x]	Flow cytometry
[x]	[ ]	MRI-based neuroimaging

Antibodies

Antibodies used	We include a table of all reagents and antibodies with sources and catalog numbers
Validation	All antibodies and reagents were purchased from well-established and reputable companies, all of which have ISO certification

Animals and other research organisms

Policy information about studies involving animals; ARRIVE guidelines recommended for reporting animal research, and Sex and Gender in Research

Laboratory animals	C57BL/6, Gsdme-/- and Nlrp3-/- mice were purchased from the Jackson Laboratories. Gsdmd-/- and Nlrc4-/- mice were a kind gift from Dr. Russell Vance (University of California, Berkeley). Gsdmd-/-/Gsdme-/- mice were generated in-house. All transgenic mice were on a C57BL/6 background. As mice were bred in-house, we used approximately equal numbers of males and females, and ordered male and female mice from Jackson Laboratories.
Wild animals	N/A
Reporting on sex	We did not account for sex differences in mice that were used only as a source of primary cells. For infection studies, we used males and females, and found no difference between them .
Field-collected samples	N/A
Ethics oversight	Animals were housed in pathogen free conditions in microisolator cages and were treated according to institutional guidelines following approval by the University of California, Irvine IACUC.

Note that full information on the approval of the study protocol must also be provided in the manuscript.

Flow Cytometry

Plots

Confirm that:

- [x] The axis labels state the marker and fluorochrome used (e.g. CD4-FITC).
- [x] The axis scales are clearly visible. Include numbers along axes only for bottom left plot of group (a 'group' is an analysis of identical markers).
- [x] All plots are contour plots with outliers or pseudocolor plots.
- [x] A numerical value for number of cells or percentage (with statistics) is provided.

Methodology

Sample preparation	Corneas from infected mice were dissected and incubated with 3 mg/ml collagenase. Cells were recovered following centrifugation and incubated with anti-mouse CD16/32 Ab (BioLegend) to block Fc receptors, then 20 min with anti-mouse CD45-allophycocyanin, Ly6GBV510, Ly6C-PE-Cy7, CD11b-PETxRed, CCR2-BV421, or F4/80-FITC (BioLegend) and fixable viability dye (BD Biosciences).
Instrument	ACEA Novocyte flow cytometer
Software	NovoExpress software
Cell population abundance	Ly6G+ neutrophils comprised ~90% total CD45+ cells in infected corneas.
Gating strategy	The gating strategy identified total cells in infected corneas by forward and side scatter, followed by gating on single cells and live cells were identified using the E780 viability dye (Biolegend). Neutrophils were identified as CD45+, CD11b+, Ly6G+, CCR2-, and monocytes were CD45+, CD11b+, Ly6G- CCR2+. Gating strategy is shown in Figure S3K.

- [x] Tick this box to confirm that a figure exemplifying the gating strategy is provided in the Supplementary Information.
